# Recent advances in smart stimuli-responsive biomaterials for bone therapeutics and regeneration

**DOI:** 10.1038/s41413-021-00180-y

**Published:** 2022-02-23

**Authors:** Hongpu Wei, Jinjie Cui, Kaili Lin, Jing Xie, Xudong Wang

**Affiliations:** 1 Department of Oral & Cranio-Maxillofacial Surgery, Shanghai Ninth People’s Hospital, College of Stomatology, Shanghai Jiao Tong University School of Medicine, National Clinical Research Center for Oral Diseases, Shanghai Key Laboratory of Stomatology & Shanghai Research Institute of Stomatology, Shanghai, 200011 China; 2 State Key Laboratory of Oral Diseases, West China Hospital of Stomatology, Sichuan University, Chengdu, 610041 China

**Keywords:** Diseases, Bone

## Abstract

Bone defects combined with tumors, infections, or other bone diseases are challenging in clinical practice. Autologous and allogeneic grafts are two main traditional remedies, but they can cause a series of complications. To address this problem, researchers have constructed various implantable biomaterials. However, the original pathological microenvironment of bone defects, such as residual tumors, severe infection, or other bone diseases, could further affect bone regeneration. Thus, the rational design of versatile biomaterials with integrated bone therapy and regeneration functions is in great demand. Many strategies have been applied to fabricate smart stimuli-responsive materials for bone therapy and regeneration, with stimuli related to external physical triggers or endogenous disease microenvironments or involving multiple integrated strategies. Typical external physical triggers include light irradiation, electric and magnetic fields, ultrasound, and mechanical stimuli. These stimuli can transform the internal atomic packing arrangements of materials and affect cell fate, thus enhancing bone tissue therapy and regeneration. In addition to the external stimuli-responsive strategy, some specific pathological microenvironments, such as excess reactive oxygen species and mild acidity in tumors, specific pH reduction and enzymes secreted by bacteria in severe infection, and electronegative potential in bone defect sites, could be used as biochemical triggers to activate bone disease therapy and bone regeneration. Herein, we summarize and discuss the rational construction of versatile biomaterials with bone therapeutic and regenerative functions. The specific mechanisms, clinical applications, and existing limitations of the newly designed biomaterials are also clarified.

## Introduction

Tumors, severe infection, osteoporosis, osteonecrosis, and some congenital malformations can cause large bone defects, which remain challenging in clinical practice.^1^ Bone defects induced by various causes are currently one of the principal causes of morbidity and disability, which decrease quality of life and endanger lives.^2^ The annual medical cost and lost wages for individuals with musculoskeletal diseases reached $849 billion in 2004, which is 7.7% of the GDP of the United States.^3^ With the increasing number of patients in an aging society, the total direct cost of patients with musculoskeletal disease increased by 117% between 2009 and 2011. Moreover, with increasing social demand, research and development costs are also rising year by year. According to Food and Drug Administration data, the clinical development cost of products in phases I–III increased to $14.0 billion in 2020. Moreover, the market for bone substitutes was estimated at over 2.3 billion US dollars in 2015, and this value was expected to exceed 3.6 billion US dollars from 2016 to 2022.^4^ The traditional treatments for bone defects include autologous and allogenic grafts, which have many disadvantages, such as secondary injury, limited resources, the risk of infectious disease, and immunological rejection.^5–7^ Other treatment methods, including distraction osteogenesis, growth factor loading, electrical stimulation, Masquelet-induced membrane technique, and various combinations of these methods, have also been developed in recent years.^8,9^ However, the tedious and arduous treatment period and numerous potential complications limit their clinical applications.

Bone tissue engineering (BTE) is a newly developed alternative approach that introduces an exogenous scaffold to encourage cells to grow and proliferate by providing regulatory growth factors.^7,8,10,11^ These implantable scaffolds are required to match the properties of the original tissues, such as osteoinductivity, biocompatibility, osteoconductivity, and suitable mechanical strength.^12,13^ Various artificial implantable scaffolds, including metal, bioceramic, biopolymer, and composite implants, are manufactured for bone tissue regeneration or BTE applications.^6,14–16^ Although bone regeneration shows excellent promise,^8^ BTE still has some fatal problems, such as high cost, the potential tumorigenic risk of growth factors, and the inability to develop a natural combination with the surrounding normal tissue.^17^ Moreover, implant fractures caused by inflammation, loosening, and osteolysis remain as challenges to be addressed.^18^

In addition, residual tumor tissue and severe infection can lead to recurrence, metastasis, or sepsis, which can ultimately endanger life. Moreover, residual tumor tissue and infection promote the release of inflammatory factors and activate osteoclasts, leading to the consumption of bone tissue and ultimately to the failure of bone regeneration.^19^ Hence, traditional resection and reconstruction cannot solve the problem entirely due to the limited ability to achieve correct autoregeneration.^6,20^ In addition to surgical resection, other standard methods, such as radiation therapy and chemotherapy, have low selectivity for tumor cells and high toxicity to normal cells, significantly limiting their clinical applications.^21^ Based on this, the rational design and construction of versatile biomaterials with both disease therapy and bone regeneration functions are in great demand.^22^

**Fig. 1:**
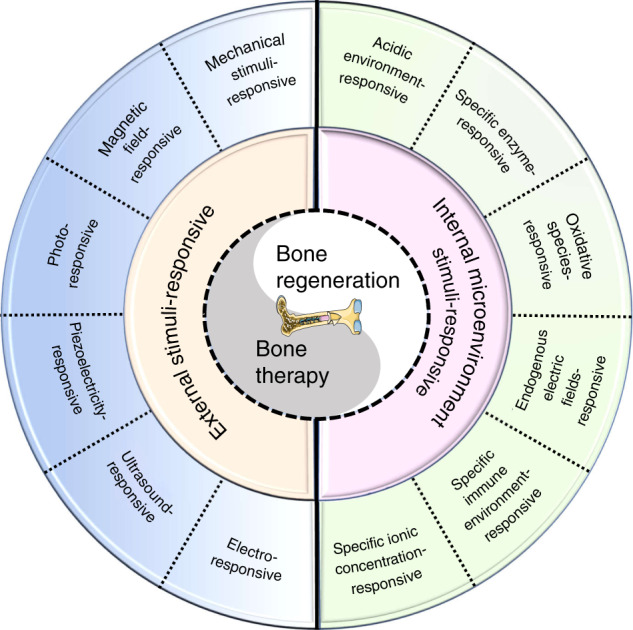
Scheme summarizing different strategies in the design and fabrication of versatile biomaterials with both therapeutic and regeneration functions

In addition, various diseases have different specific pathological microenvironments, such as excess reactive oxygen species (ROS) and specific mild acidity in tumors,^23,24^ specific pH reduction and enzymes secreted by bacteria in severe infection,^25–27^ and electronegative potentials in bone defect sites.^28^ Based on these specific pathological features, researchers designed corresponding materials that can autonomously respond to the specific environmental changes surrounding the lesion, thus activating bone disease therapy and bone defect regeneration. Such design strategies were defined as internal microenvironment stimuli-responsive strategies in this review. In addition, external physical stimuli, such as light, magnetic fields, electricity, ultrasound, or appropriate mechanical stimuli, can transform the internal atomic packing arrangements of materials, thus affecting cell fate and enhancing bone tissue therapy and regeneration.^29,30^ The design strategies utilizing external physical stimuli were defined as external stimuli-responsive strategies in this review. Based on these concepts, a number of novel bifunctional materials with smart stimuli-responsive therapeutic and regenerative abilities were developed,^31,32^ which could undergo reversible or irreversible transformations in physical performance or chemical structure in response to external physical triggers (e.g., light irradiation, electric and magnetic fields, ultrasound, appropriate mechanical stimulus), endogenous disease microenvironments (e.g., overexpressed ROS, mild acidity, endogenous electric fields, specific ionic concentrations, secreted enzymes or specific immune environments), or a combination of the above (Fig. 1).^22,33,34^

The definition of “smart” here refers to the properties of biomaterials that can exert stimulating or inductive effects on tissues by responding to external or internal stimuli.^3^ Smart on-demand stimuli-responsive biomaterials can maximize therapeutic efficacy and minimize undesirable side effects because they can rapidly detect and respond to the disease environment and exert therapeutic effects while preserving physiologically healthy cells and tissues, thus improving patient quality of life.^35,36^ Recently, Montoya et al.^37^ proposed a redefinition of the term “smart biomaterial” to recognize four levels of smartness: inert, active, responsive, and autonomous. Their well-organized review also divides smart biomaterials into two categories, namely, smart biomaterials that respond to internal material properties (e.g., topography, mechanical properties, surface charge, and scaffold chemistry) and smart biomaterials that respond to external stimuli (e.g., piezoelectricity, magnetism, pH, and enzymes). This classification would definitely facilitate the construction of new material systems and the exploration of potential mechanisms. However, the range of external stimuli mentioned in this review might be too broad. Accordingly, we further subdivide it into two parts, namely, an internal microenvironment stimuli-responsive strategy and an external stimuli-responsive strategy. Moreover, some well-constructed multifunctional biomaterials apply multiple strategies to achieve outstanding effects. Thus, multiresponsive strategies are also addressed in our review.

**Fig. 2:**
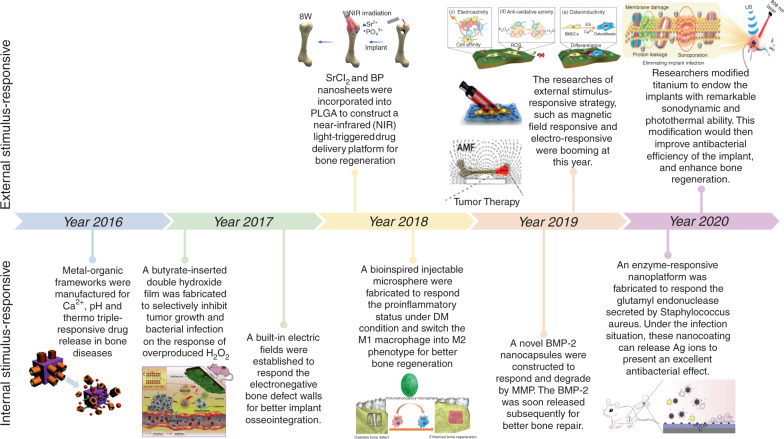
Timeline of some representative studies of the application of smart stimuli-responsive biomaterials in the past half-decade. Figures for years 2016–2020: Image for 2016: reproduced with permission.^115^ Copyright 2016, Royal Society of Chemistry; Image for 2017: reproduced with permission.^104^ Copyright 2017, Elsevier; Image for 2018: reproduced with permission.^19^ Copyright 2018, Elsevier; reproduced with permission.^155^ Copyright 2018, American Chemical Society; Image for 2019: reproduced with permission.^54^ Copyright 2019, Elsevier; reproduced with permission.^61^ Copyright 2019, American Chemical Society; reproduced with permission.^77^ Copyright 2019, Wiley-VCH; Image for 2020: reproduced with permission.^31^ Copyright 2020, American Chemical Society; reproduced with permission.^126^ Copyright 2020, Royal Society of Chemistry

Smart stimuli-responsive biomaterials differ from traditional biomaterials due to their response to stimulating/triggering factors in the surrounding environment (both external and internal).^38,39^ Thus, these novel biomaterials have received increasing attention from researchers in recent years. Rapid progress in the manufacture of smart stimuli-responsive biomaterials has occurred in the past 5 years, from applying external stimulation to enhance bone regeneration and therapeutic effects for smartly responses to variations in the internal microenvironment to rational synergistic therapy combining multiple strategies to improve therapeutic efficacy (Fig. 2). To summarize the previous research results and facilitate the further design and application of these novel smart stimuli-responsive materials for bone tissue therapy and regeneration in the biomedical field, we summarize the recent advances in smart stimuli-responsive biomaterials in this field. In this review, we summarize the different stimuli-responsive strategies, both external stimuli-responsive strategies and internal microenvironment stimuli-responsive strategies, and illustrate the classical biomedical applications for bone therapy and regeneration in recent studies. We also compare the pros and cons of different strategies, and we discuss the current challenges and future prospects of these novel biomaterials. This knowledge might assist in constructing versatile biomaterials in the future to meet the demand for bone regeneration in various environments.

## External stimuli-responsive bone therapy and regeneration

External stimuli such as light, magnetic fields, ultrasound, electrical stimulation, and appropriate mechanical stimuli can generate heat or stimulate bone cells to adhere, proliferate, and differentiate in scaffolds, thus facilitating bone therapy and regeneration.^8,29^ In addition, most therapeutic biomaterials are constructed from nanomaterials with therapeutic functions, such as magnetic nanoparticles (NPs) for magnetothermal ablation, photothermal nanoagents for photonic hyperthermia or drug nanocarriers for chemotherapy.^22,40–42^ Thus, integrating these therapeutic NPs into bone regeneration scaffolds to construct smart stimuli-responsive scaffolds for bone therapy and regeneration is a promising and encouraging approach. A multitude of studies have focused on this field and fabricated many novel biomaterials based on these strategies. Herein, we have summarized and illustrated various kinds of external stimuli-responsive strategies in the following section.

### Photoresponsive strategy

Radiation under an infrared laser can generate photophysical effects, subsequently changing intracellular behavior and influencing the respiratory chain, which ultimately increases ATP regeneration in the mitochondrial membrane and heightens cell metabolism.^43^ These effects could facilitate angiogenesis and bone regeneration.^44^ Therefore, the photoresponsive strategy is widely applied in both antitumor and antibacterial treatment due to easy synthesis and the presence of plentiful photoresponsive functional nanosystems and components.^22,45,46^ Common photothermal agents include gold nanostructures, transition metal sulfides and oxides (e.g., CuFeSe_2_ nanocrystals, Fe_3_O_4_ NPs, and copper silicate microspheres), organic NPs, carbon-based NPs and graphene, MXenes, and single-elemental nanosheets (e.g., black phosphorus nanosheets (BPs)).^47–53^

**Fig. 3:**
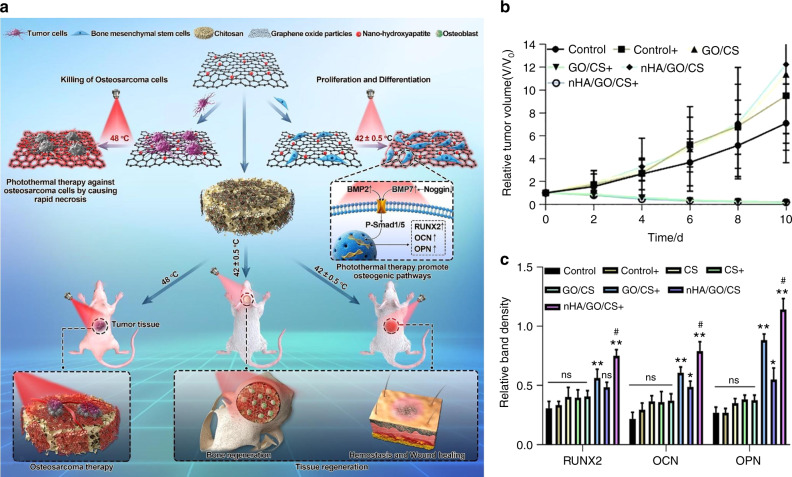
nHA/GO/CS scaffolds for both disease therapeutic and bone regeneration. **a** Scheme to manifest the fabrication of nHA/GO/CS scaffolds with bifunctionalities of both therapeutic and regeneration. **b** Tumor volume changes after different treatments with time (days). **c** Quantitative analyses of various proteins with or without NIR exposure after 14 days of osteogenic culture (**P < 0.01, *P < 0.05). Reproduced with permission.^55^ Copyright 2020, Elsevier

Many studies have focused on this strategy and synthesized numerous multifunctional materials due to encouraging results. Tong et al.^54^ recently directly integrated BP nanosheets into poly(lactic-co-glycolic acid) (PLGA) to construct a multifunctional scaffold (designated BPs@PLGA) for efficient osteogenesis. In this degradable biocomposite, BPs@PLGA containing 0.2 wt% BPs exhibited highly efficient photoresponsive osteogenesis when covered by biological tissue. When exposed to near-infrared (NIR) light, the BPs@PLGA specimen can promote osteogenesis by upregulating heat shock protein expression. Similarly, Wang et al.^19^ constructed BP-SrCl_2_/PLGA microspheres by directly loading BPs and SrCl_2_ into PLGA. NIR radiation can trigger the release of Sr^2+^, which can ultimately improve bone regeneration, since the local temperature increase creates flaws in the PLGA shells. Similarly, to achieve radical tumor ablation and new bone regeneration for osseous tumor therapy, nanohydroxyapatite/graphene oxide (nHA/GO) particles were used to functionalize chitosan (CS) scaffolds for their outstanding photothermal conversion performance and bone-forming bioactivity (Fig. 3a).^55^ Under 808-nm NIR irradiation, the temperature was increased to 48 °C, which effectively ablated human osteosarcoma cells (Fig. 3b). Moreover, NIR irradiation may enhance the BMP-2/Smad signaling pathway, which can significantly promote the osteogenesis of hBMSCs (Fig. 3c).

**Fig. 4:**
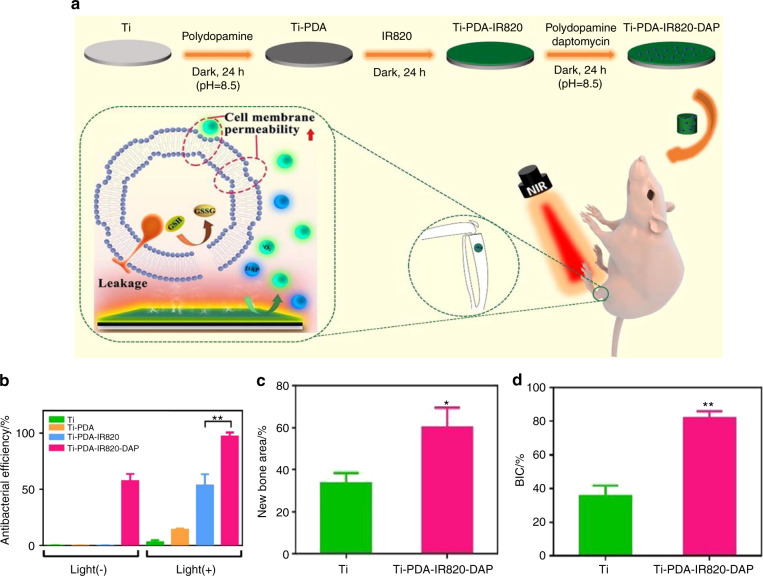
A novel biocompatible PDA/IR820/DAP coating for antibiotic/photodynamic/photothermal triple therapy. **a** Scheme of the antibacterial mechanism of Ti-PDA-IR820-DAP: the synergistical therapy of DAP, PTT, and PDT cause remarkable lethal effect to bacteria. **b** The results of spread plate assays to show the antibacterial efficiency. **c** Quantitative analysis of new bone area. **d** The percentages of bone-implant contact (BIC) were calculated from Van Gieson staining. Reproduced with permission.^56^ Copyright 2020, Elsevier

In addition to the encouraging results in osseous tumor therapy, a photoresponsive strategy was introduced to solve the difficult issue of surgical site infection. Zeng et al.^56^ fabricated a biocompatible polydopamine (PDA)-IR820-daptomycin (DAP) coating in titanium (Ti) implants, which possesses the triple therapeutic functions of antibiotic, photodynamic, and photothermal activity (Fig. 4a). Laser irradiation at 808 nm can stimulate PDA hyperthermia and release the singlet oxygen (^1^O_2_) generated by IR820, which can eradicate biofilms noninvasively. Furthermore, the successful acceleration of glutathione oxidation, release of DAP, and destruction of the bacterial membrane can synergistically eliminate the target bacteria with an antibacterial efficiency of 97.2% (Fig. 4b). Furthermore, the novel PDA coating increased the surface roughness, thus upregulating osteogenic-related gene expression and attaining a higher bone-implant contact level, which ultimately enhanced the bone regeneration ability (Fig. 4c, d).

In addition to the encouraging results of the photoresponsive biomaterials described above, several issues still need to be settled in the future. The low tissue penetration depth of light severely limits the ability of photothermal conversion, thus ultimately impeding deep tissue regeneration in the body. Thus, the exploration of other controllable and noninvasive physical triggers to improve tissue penetration capability is desperately needed in the future to accelerate the clinical translation of these novel strategies.^22,43^

### Magnetic field-responsive strategy

Magnetic NPs, usually referred to as Fe_3_O_4_ NPs, can serve as therapeutic agents for magnetic hyperthermia, not only generating heat under exposure to an external magnetic field but also improving the osteogenic differentiation ability, thus making this technology highly promising in bone tissue regenerative applications.^22,57,58^ After exposure to irradiation with an external magnetic field, the heat produced by magnetic Fe_3_O_4_ NPs can elevate the temperature to 42 °C–45 °C, which can damage or even kill cancerous cells due to hemorrhage or vascular occlusion while remaining harmless to the normal surrounding tissues.^24,59^ In addition, magnetic hyperthermia might also contribute to inducing better osteogenic differentiation. Nevertheless, the potential mechanisms remain unclear. Some researchers hold the view that hyperthermia could influence bone metabolism by enhancing the blood supply.^57^ Other researchers believe that hyperthermia improves osteogenic expression by enhancing mitochondrial activity and accelerating the expression of bone-related genes.^60^ The related detailed mechanisms for the osteogenic stimulation derived from magnetic hyperthermia still need to be further elucidated. Compared to the photoresponsive strategy, magnetothermal therapy by an external alternating magnetic field has the advantage of high tissue penetration capability, which make it promising for treating lesions in deep tissue, such as bone tumors. Moreover, magnetic field-responsive therapy has the advantages of noninvasiveness and high controllability, which are in high demand for bone tumor ablation and local bone regeneration.

**Fig. 5:**
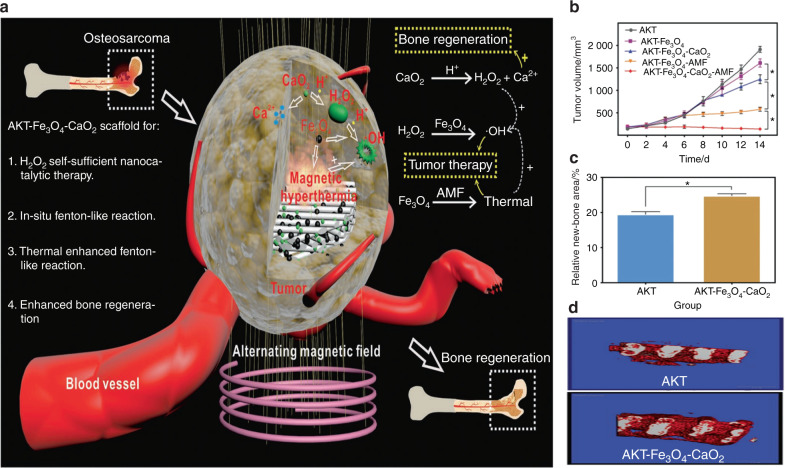
3D-printing scaffolds coloading with Fe_3_O_4_ and CaO_2_ NPs (AKT-Fe_3_O_4_-CaO_2_) for cancer therapeutic and bone regeneration. **a** Scheme of the fabrication of 3DP AKT-Fe_3_O_4_-CaO_2_ scaffold with bifunction of magnetic hyperthermia and bone regeneration. **b** Time-dependent tumor-volume changes of mice after different treatment (*P < 0.05). **c** Quantitative analysis of newborn bone tissues after VG stained. **d** 3D reconstruction of micro-CT images showing the in vivo osteogenesis performance directly (red, newborn bone tissues; white, residual scaffolds). Reproduced with permission.^24^ Copyright 2020, Wiley-VCH

Many researchers have exploited versatile multifunctional biomaterials using a magnetic field-responsive strategy, combining the functions of bone disease therapy and bone tissue regeneration. Zhu et al.^58^ fabricated magnetic 10Fe5Ca MBG scaffolds (Fe_3_O_4_–CaO–SiO_2_–P_2_O_5_ system), which could produce heat upon exposure to an external alternating magnetic field. In addition, osteoblast cell proliferation, alkaline phosphatase (ALP) activity, and osteogenic expression could also be enhanced because of the lower ion dissolution rate and the possibility of maintaining a beneficial pH value. In the lower pH environment, drugs such as gentamicin were easily released, resulting in a corresponding therapeutic effect. With the multifunctionality of magnetic hyperthermia, local drug delivery therapy, and bone regeneration, these synthetic magnetic scaffolds have high future potential in the treatment of bone tumors. Similarly, Dong et al.^24^ integrated calcium peroxide (CaO_2_) and iron oxide (Fe_3_O_4_) NPs with an akermanite (AKT) scaffold (designated AKT-Fe_3_O_4_-CaO_2_) using a three-dimensional printing (3DP) technique to combine magnetic hyperthermia and bone regeneration. The loaded Fe_3_O_4_ NPs not only served as triggers to initiate magnetic hyperthermia for quick temperature elevation but also acted as nanocatalysts to initiate the Fenton reaction in tumor-oxidative therapy (Fig. 5a). In addition, the loaded CaO_2_ NPs can react to the acidic tumor environment and subsequently produce H_2_O_2_, which could not only remedy the consumption of H_2_O_2_ via the Fenton reaction but also release Ca^2+^ ions to further induce bone regeneration. The incorporation of nanocatalytic oxidative therapy and magnetic hyperthermia significantly restrained tumor growth compared to either single therapy (Fig. 5b). In addition, the continuous release of Ca^2+^ within the scaffolds obviously improved osteogenesis, as shown by both micro-CT reconstruction and quantitative analysis of Van Gieson’s (VG) staining techniques (Fig. 5c, d). Similarly, Yan et al.^61^ constructed an injectable magnetic bone cement (α-tricalcium phosphate (α-TCP)/CS/Fe_3_O_4_/GO, αCFG) by loading Fe_3_O_4_/GO nanocomposites into α-TCP/calcium sulfate biphasic bone cement. The αCFG bone cement with 10 wt % Fe_3_O_4_/GO content was the most rigid and showed remarkable magnetothermal performance. Bone regeneration ability might also be enhanced by promoting the attachment, proliferation, and osteogenic differentiation of rBMSCs.

Although all the studies related to magnetic field-triggered bone therapy and regeneration are at a preliminary stage, the encouraging results are capturing increasing attention. Further research may concentrate on uniform magnetic heating and methods for reducing the risk of thermal damage to surrounding normal tissues.^61^

### Ultrasound-responsive strategy

In addition to photodynamic therapy (PDT), sonodynamic therapy (SDT) is a highly promising noninvasive technique for eradicating tumor cells. In contrast to PDT, SDT is initiated by ultrasound with a tissue penetration depth of over 10 cm, which has been widely applied in clinical practice for several years in ablating deep-seated tumors.^62^ In addition to tumor ablation, ultrasound was proven to enhance bone regeneration since it could enhance the mRNA level of vascular endothelial growth factor A (VEGF-A) and stimulate cartilage cell proliferation, thus accelerating the maturation of newly formed bone and expediting cell mineralization.^63,64^ With in-depth research, this technique was extended to the infection field for the clinical treatment of bone defects and severe bacterial infection.^27,31,65^ Crasto et al.^66^ fabricated novel liposome-rhBMP-2 nanocomplexes that can release rhBMP-2 after exposure to nonthermogenic clinical diagnostic ultrasound. After implantation into the hindleg muscles, the liposome-rhBMP-2 nanocomplexes induce local bone formation only after ultrasound exposure. Moreover, further research showed that rhBMP-2 release behavior varied with the duration of exposure and applied ultrasound pressure.

**Fig. 6:**
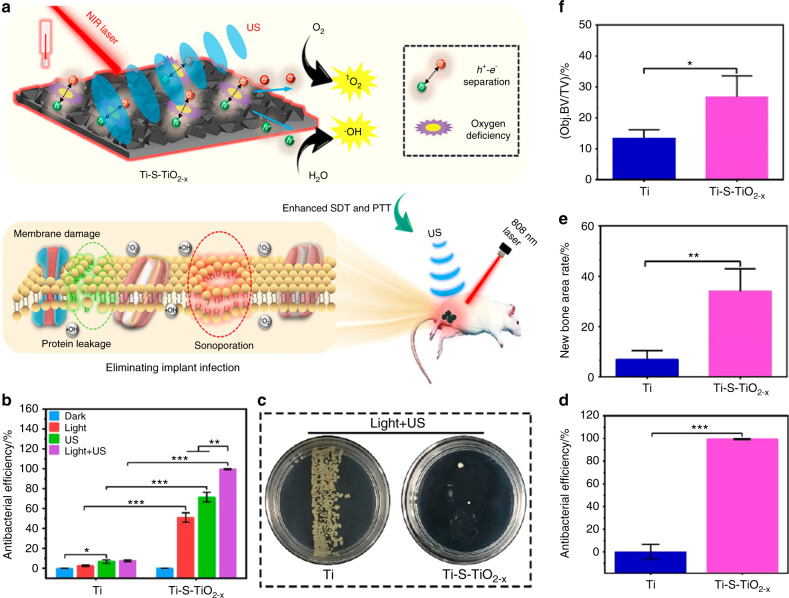
Rapid photo-sonotherapy for clinical treatment of bacterial infected bone implants by creating oxygen deficiency using sulfur doping. **a** Scheme of the fabrication of sulfur-doped om titanium implant (Ti-S-TiO_2–*x*_), which can enhance sonocatalytic-photothermal ability and manifest exhibits efficient bone infection therapy. **b** In vitro antibacterial efficiency of 2 kinds of titanium implant in four different conditions for S. aureus from spread plate. **c** Bacteria colony images to reveal the in vivo antibacterial performance. **d** The results of the spread plate to reveal the in vivo antibacterial efficiency. **e** Corresponding calculated new bone area to reveal the in vivo osteogenic performance. **f** The results of bone volume/total volume (BV/TV) based on the micro-CT results (*P < 0.05, **P < 0.01, ***P < 0.001). Reproduced with permission.^31^ Copyright 2020, American Chemical Society

To achieve better sonodynamic and photothermal ability, Su et al.^31^ modified Ti implants through sulfur (S) doping to create an oxygen deficiency (Ti-S-TiO_2-*x*_), endowing this implant with remarkable sonodynamic and photothermal ability (Fig. 6a). Without an external antibacterial coating, the novel Ti implant can reach an antibacterial efficiency of up to 99.995% against *Staphylococcus aureus* under 15 min of ultrasound and NIR light exposure (Fig. 6b–d). After combined therapy, the severe bone infection was successfully treated, and the osseointegration was improved (Fig. 6e, f). Similar to the photoresponsive strategy, this ultrasound-responsive strategy is noninvasive with high controllability. Moreover, compared to traditional laser exposure, this unique method has high tissue penetration capability, which makes it feasible for the therapy of deep-seated diseases.

### Electroresponsive strategy

Electrical stimulation has been demonstrated to accelerate bone regeneration and maintain bone marrow mesenchymal stem cell (BMSC) stemness both in animal experiments and in clinical practice.^67–70^ Electrical stimulation resulted in significant new bone formation because mesenchymal stem cell proliferation and differentiation were stimulated.^71^ This underlying mechanism contributes to upregulating bone morphogenetic proteins under electrical stimulation, thus ultimately stimulating the calcium–calmodulin pathway, transforming growth factor-β (TGF-β) and other cytokines.^67,68,72^ Loading electroactive materials, such as carbon nanotubes, metal, graphene, inorganic electroactive materials, and conductive polymers, is a direct way to deliver and respond to localized electrical stimulation and thus will better regulate cellular activities and achieve better regeneration effects.^73^ Among all these electroactive biomaterials, conducting polymers, such as polypyrrole (PPy), polyaniline (PANI), and poly(3,4-ethylenedioxythiophene), have conductivities similar to those of metals and inorganic semiconductor materials. They also have the advantages of good biocompatibility and ease of synthesis.^67,74–76^ Thus, these kinds of conducting polymers have been widely applied in biomedical tissue engineering field.^73^

**Fig. 7:**
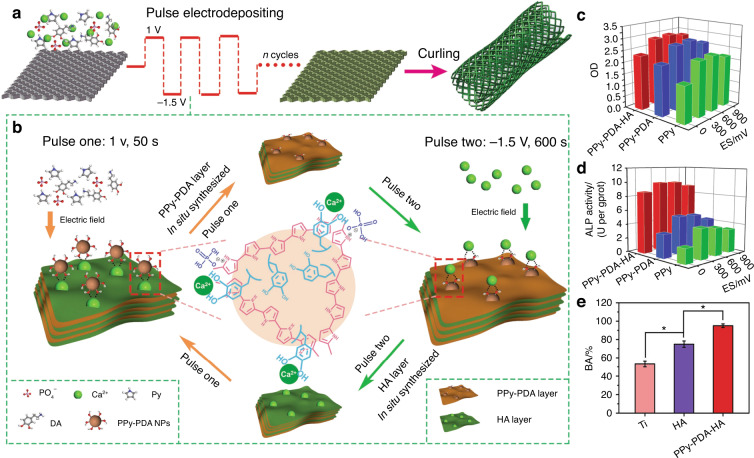
A mussel-inspired persistent ROS-scavenging, electroactive, and osteoinductive scaffold based on electrochemical-driven in situ nanoassembly. **a** Scheme of the fabrication of PPy-PDA-HA-coated scaffolds by curling after coating a PPy-PDA-HA film on a titanium mesh. **b** Fabrication of the PPy-PDA-HA film by layer-by-layer pulse electrodeposition (LBL-PED) method. **c** proliferation, and **d** differentiation of BMSCs on the PPy, PPy-PDA, and PPy-PDA-HA films under different electrical stimulation potentials. **e** Comparison of bone area (BA) from histomorphometry. Reproduced with permission (*P < 0.05).^77^ Copyright 2019, Wiley-VCH

A multitude of multifunctional materials that utilize this strategy to enhance osteogenesis expression were fabricated recently. Zhou et al.^77^ integrated PPy-PDA NPs with HA NPs using a layer-by-layer pulse electrodeposition (LBL-PED) method. After that, the PPy-PDA-HA film was uniformly coated on the Ti surface to fabricate an osteoinductive, cell affinitive, and electroactive porous Ti scaffold (Fig. 7a, b). When electrical stimulation was applied, the conductive polymer activated the Ca^2+^ channel of the cell membrane, thus facilitating extracellular Ca^2+^ entry into cells and subsequently activating the Ca^2+^ signal transduction pathway. All these changes upregulated the expression of related genes. Ultimately, with the combined effect of HA and electrical stimulation, osteogenic cell differentiation was promoted significantly (Fig. 7c–e), which illustrated its potential application as an implant for bone regeneration.

In addition, to achieve both antibiotic and osteogenic therapy, Zhu et al.^76^ recently applied Ag-loaded poly(amide-amine) dendrimer as the dopant in PANI fabrication and then coated Ti sheets with this multifunctional coating. This PANI (PAMAM)@Ag layer coating combined with electrical stimulation synergistically enhanced osteogenesis expression. In addition, these unique coatings exhibited remarkable antibacterial properties 1 000 times greater than those of pure Ti. Similarly, GdPO_4_·H_2_O nanobundles were used to functionalize PLGA and poly(3-hexylthiophene) (P3HT) nanocomposites for electrically and magnetically responsive bone regeneration.^73^ The multifunctional GdPO_4_·H_2_O (7.0 wt%)/P3HT/PLGA composite could apparently increase calcium deposition and ALP activity and upregulate the expression of related genes, thus enhancing osteogenic differentiation. Under electrical stimulation at 500 mV and 100 Hz, enhanced osteogenic differentiation was observed. Moreover, the composites were traceable by X-ray and MRI owing to the incorporation of GdPO_4_·H_2_O nanobundles. Therefore, this novel biomaterial, which exhibits favorable electroactivity, biocompatibility, and traceability, may be widely used to trace bone fixing materials and bone implants, providing a noninvasive method to observe implants.

Despite the encouraging effect of electrical stimulation on osteogenesis, certain issues still need to be settled before large-scale clinical application. The long-term cytotoxicity, biocompatibility, and biodegradability of conductive scaffolds in vivo need to be further researched due to their long-term presence in vivo. We believe that conductive biomaterials will definitely become new tissue engineering materials with great potential in the future.

### Piezoelectricity-responsive strategy

Natural living bone tissue has bioelectrical properties, which play a critical role in bone development.^75,78^ Piezoelectricity is defined as the phenomenon of transforming mechanical energy into electrical energy.^78^ Under physiological conditions, bone can generate electrical signals when stressed by mechanical stimuli. These electrical signals can ultimately promote bone growth and remodeling.^79^ This unique phenomenon was observed by researchers and innovatively applied in developing novel biomaterials. These newly developed biomaterials were defined as piezoelectric materials, which are a class of smart mechanoelectrical transducers that can generate electrical signals under stress.^80^ It has been proven that piezoelectricity can regulate cell function and benefit the proliferation and osteogenic differentiation of stem cells. Therefore, piezoelectric biomaterials are gradually attracting attention in the field of bone healing and BTE.^81,82^ Representative piezoelectric biomaterials, including piezo-bioceramics (e.g., barium titanate (BaTiO_3_), magnesium silicate, zinc oxide) and some piezo-biopolymers (e.g., polyvinylidene fluoride, polyhydroxybutyrate, etc.), have been widely applied in biomedical fields.^83,84^

On the basis of previous studies on piezoelectric materials, attaching piezoelectric materials to enhance piezoelectric properties has become an effective method in BTE applications. To achieve a better bone regeneration effect, Tang et al.^85^ fabricated HA/BaTiO_3_ composite materials via slip casting. Subsequent polarization endowed the composite materials with piezoelectric properties. After being subjected to cycle loading, the piezoelectric effect of BaTiO_3_ could facilitate the growth of osteoblasts and promote interaction with HA. This rational design would provide a new method for bone defect regeneration. Contemporaneously, Bai et al.^86^ utilized this strategy in classic guided bone regeneration therapy to address the limitation of insufficient osteoinductivity in currently available barrier membranes. The flexible nanocomposite membrane was fabricated by homogeneously distributing piezoelectric BaTiO_3_ NPs into a poly(vinylidene fluoridetrifluoroethylene) matrix. This unique technology achieved higher osteoinductivity and induced earlier neovascularization. After implantation with DBB Bio-Oss^®^ granules, mature bone structure formation was observed in critical-sized defect sites in rabbit mandibles treated with this novel design. Recently, Zhao et al.^87^ constructed a periosteum structure/function-mimicking scaffold by rationally integrating poly(vinylidene fluoride-trifluoroethylene) (PVFT) and bioactive glass. The gradient structure of this rational design includes a bioglass nanofibrous surface and a piezoelectric polymer layer for synergistically enhancing bone regeneration and periosteum formation in critical-sized bone defects. The biomimetic scaffolds significantly improved the proliferation, migration, and osteogenic differentiation of mBMSCs, thus improving the formation of periosteum-like tissue and facilitating bone regeneration in critical-sized defects. In addition, the negative pole of the PVFT layer can gather positive Ca^2+^ from bioactive glass, activating the CaSR of osteoblasts and ultimately enhancing osteogenesis. This unique work presents a novel choice in applying biomimetic piezoelectric behavior for efficient bone regeneration in critical-sized bone defects.

In general, piezoelectricity-responsive biomaterials provide a natural physiological environment to regulate stem cell functions without extraneous drugs or growth factors, which could serve as an innovation in bone regeneration and BTE. However, some limitations still remain to be addressed, such as the effects of densification, alkali volatilization, and high temperature in the synthesis processing.^84^ In addition, the potential mechanisms and long-term biosafety and toxicity also remain to be explored in the future. There is still a long way to go before the ultimate clinical translation of this novel strategy.

### Mechanical stimuli-responsive strategy

Osteocytes are mechanosensitive cells, and it has been widely proven that mechanical stimuli could influence stem cell fate, including proliferation, migration, and differentiation.^88,89^ The proper mechanical stimulus has been proven to play a vital role in the metabolism and repair of new bone formation.^90^ The lack of proper mechanical stimulus in new bone would reduce the expression of related osteogenic genes, thus impeding further new bone formation.^91^ However, the improper magnitude of strains may lead to bone nonunion, even bone resorption.^92^ Numerous studies have focused on this phenomenon and attempted to explore its underlying mechanism. The transcription factors YAP and TAZ were demonstrated to play significant roles in this process.^93^ YAP and TAZ can read a wide range of mechanical cues, such as extracellular matrix rigidity and shear stress, and translate them into unique transcriptional programs with further essential regulatory roles.^93^ In addition, researchers found that continuous mechanical strain can rapidly activate the PI3K/Akt signaling pathway and prompt maximum phosphorylation in a short duration. These transformations would induce the early differentiation of BMSCs toward an osteogenic phenotype, thus regulating bone formation and improving bone regeneration even in the osteoporosis status.^94^ Furthermore, Eichholz et al.^95^ demonstrated that when affected by fluid shear, osteocytes could secrete many distinct factors to promote hMSC recruitment and osteogenesis. This finding demonstrated great potential in improving bone regeneration in diseases such as osteoporosis.

Based on previous work, some researchers have started to explore incorporating this strategy into bone regeneration. Puwanun et al.^96^ used a standard see-saw rocker to generate oscillatory fluid flow in vitro. After rocking at 45 cycles per min for 1 h per day and 5 days per week, bone differentiation and vascularization were significantly enhanced, which would provide a simple and cost-effective therapy for bone regeneration in minor defects such as cleft palate. Similarly, Mohanraj et al.^97^ fabricated interesting mechanically activated microcapsules to deliver specific medicine in response to a mechanically loaded environment for the smart regeneration of musculoskeletal tissues. These novel microcapsules release TGF-β3 upon mechanical stimulation, presenting a new perspective in tissue regeneration.

However, all studies are still at the early stage of cell-level tests. Some challenges still exist before mechanical stimuli can be used to promote bone tissue regeneration. An important issue to address in future studies is the optimization of mechanical parameters, such as the patterns, amplitude, and frequency of mechanics, to fully facilitate new bone formation.^98^ Moreover, discovering a noninvasive method to apply mechanical stimuli in the regeneration process is necessary before this method can be utilized in bone tissue regeneration.^99^

## Internal microenvironment stimuli-responsive bone therapy and regeneration

Although outstanding therapeutic and regenerative effects have been obtained with external stimuli-responsive materials, several disadvantages remain to be addressed. First, the maintenance of the effect requires the continuous action of external stimuli, which is nearly impossible for the extended treatment period of bone therapy and regeneration. In addition, the intensity of external stimuli is generally positively correlated with the therapeutic effect, but side effects also increase with stimulation intensity, thus significantly limiting the therapeutic effect. Finally, external stimuli can be applied only after the disease is diagnosed, which causes a lag effect. Moreover, various diseases have different and specific pathological microenvironments, such as specific mild acidity, excess ROS, electronegative potentials, specific ionic concentrations, particular immune environments, or enzyme release. All these specific pathological features can be applied to design corresponding smart stimuli-responsive biomaterials for rapid-response bone therapy and regeneration. Different internal microenvironment stimuli-responsive strategies are summarized and illustrated in the following section.

### Oxidative species-responsive strategy

ROS, such as peroxides, hydroxyl radicals, superoxide, singlet oxygen, and alpha-oxygen, are chemically reactive chemical species containing oxygen.^100,101^ Overexpression of ROS may occur under various pathological conditions such as bodily injury, inflammation, neurodegenerative diseases, solid tumors, and cardiovascular complications.^102,103^ Thus, excess endogenous ROS are often used as a trigger in stimuli-responsive bone therapy and regeneration. A multitude of recent studies have focused on this strategy and synthesized multifunctional biomaterials applying this strategy.

**Fig. 8:**
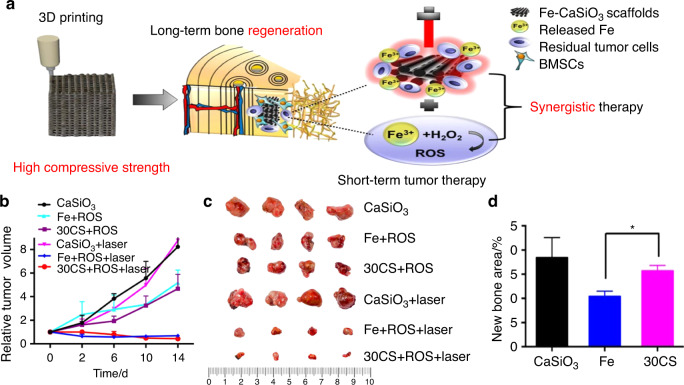
3D printing of high-strength bioscaffolds for the synergistic treatment of bone cancer. **a** Scheme of the synthesis of Fe-CaSiO_3_ composite scaffolds and their biomedical application. **b** Changes of the relative tumor volume in the six groups. **c** Photos of the tumors in six different groups on day 15. **d** The histomorphometric measurements of the in vivo new bone area in three groups at 8 weeks post-surgery (*P < 0.05). Reproduced with permission.^105^ Copyright 2018, Nature Publishing Group

To fabricate scaffolds with selective tumor-killing and bacteria-killing effects, an internal microenvironment-responsive composite scaffold was constructed by Wang et al. using a simple hydrothermal treatment.^104^ The butyrate-inserted Ni–Ti LDH film can selectively suppress bacterial infection and tumor growth by exploiting the overexpression of H_2_O_2_ in tumor and infection microenvironments, thus releasing cytotoxic butyrate to inhibit metastasis and enhance osteogenesis. Similarly, Ma et al.^105^ recently constructed optimized Fe-CaSiO_3_ composite scaffolds (30CS) using the 3DP technique (Fig. 8a). These novel scaffolds have high mechanical strength and could function in both ROS tumor therapy and photothermal therapy (Fig. 8b, c). The consumption of ROS and the presence of CaSiO_3_ enhanced the proliferation and differentiation of rBMSCs, thus enhancing osteogenesis in vivo (Fig. 8d).

ROS are often generated in a multitude of pathological processes, such as solid tumors, severe infection, cardiovascular complications, neurodegenerative diseases, and inflammatory diseases.^102^ However, ROS have the drawback of a small action range and short lifespan, which significantly hamper the stimulus effect. In addition, ROS therapies have no specificity and will damage normal cells simultaneously.

### Acidic environment-responsive strategy

**Fig. 9:**
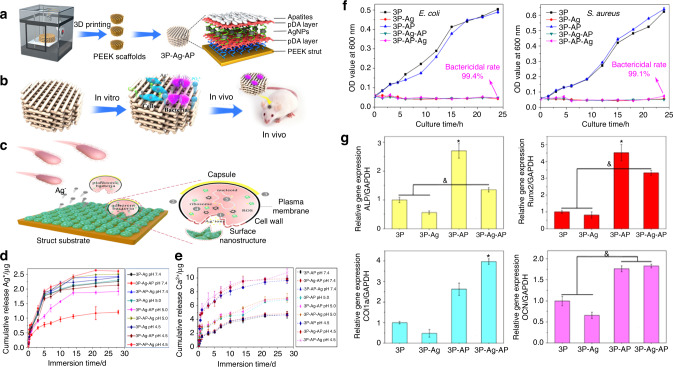
Bacteria-triggered pH-responsive osteopotentiating coating on 3D-printed polyetheretherketone scaffolds for infective bone defect repair. **a** Scheme of the fabrication of 3P-AP-Ag coatings with pH-triggered osteopotentiating properties on 3DP porous PEEK scaffolds. **b** Schematic of the tests in vitro and in vivo for their multifunction. **c** Schematic diagram of possible antibacterial factors. Three major factors might contribute to its antibacterial properties: ROS overproduction, Ag^+^ ion liberation, and surface nanostructure. **d** Delivery profiles of Ag^+^ ions from different scaffolds in various pH values (pH = 7.4, 5.0, 4.5). **e** Delivery profiles of Ca^2+^ ions from different scaffolds in various pH values (pH = 7.4, 5.0, 4.5). **f** The bactericidal curves to reveal the antibacterial activities of the coatings. **g** Quantitative reverse transcription polymerase chain reaction (RT–qPCR) analysis of the gene expressions relates to osteogenesis (ALP, Runx2, Col1a1, and OCN) (*P < 0.05, ^&^*P* < 0.05). Reproduced with permission.^26^ Copyright 2020, American Chemical Society

The microenvironment of the normal human body is weakly alkaline. However, in some specific pathological conditions, such as chronic inflammation, infected or contaminated environments, and tumor environments, the humoral environment may become mildly acidic.^106–109^ Accordingly, this microenvironmental characteristic has been exploited by some researchers to fabricate smart responsive biomaterials with both therapeutic and regenerative functions.^110^ On this basis, a composite AKT-Fe_3_O_4_-CaO_2_ scaffold was fabricated by directly incorporating calcium peroxide (CaO_2_) and iron oxide (Fe_3_O_4_) NPs into an AKT scaffold to promote simultaneous local magnetic hyperthermia and new bone formation in bone defects.^24^ The loaded CaO_2_ NPs can generate sufficient H_2_O_2_ in a mildly acidic tumor environment. Therefore, H_2_O_2_ can be catalyzed by Fe_3_O_4_ NPs to produce hydroxyl radicals (·OH), which is an ROS with high antibacterial activity and cytotoxicity. Thus, ROS can work synergistically with the therapeutic effects of magnetic hyperthermia and bone regeneration. Similarly, Deng et al.^26^ recently synthesized a unique “pDA-Ag-pDA” sandwich structure coating by trapping silver NPs on the first pDA layer and then loading apatite on the second pDA layer. These novel coatings endowed the polyetheretherketone (PEEK) scaffold with bacteria-triggered acidity-responsive ion-release behavior (Fig. 9a–c). In the acidic microenvironment of bacterial infection, Ag^+^ ions will be liberated immediately for bacterial killing (Fig. 9d, f), and Ca^2+^ and PO_4_^3−^ ions will be rapidly delivered for osteogenesis enhancement (Fig. 9e, g).

In addition to acute inflammation and tumors, some chronic diseases also manifest mild acidity in the humoral environment, which may be harmful for subsequent bone tissue regeneration. Accordingly, several smart acidic environment-responsive biomaterials have been constructed for bifunctional bone disease therapy and bone tissue regeneration. Osteoporosis is a worldwide chronic disease characterized by serious microarchitectural destruction of osseous tissue and low bone mass. Recently, Lin et al.^111^ loaded sodium bicarbonate (NaHCO_3_) into tetracycline-functionalized nanoliposomes to construct a smart “nanosacrificial layer.” This smart layer can target bone surfaces and respond to the local acidic environment caused by the abnormal activation of osteoclasts. When the constructed smart layer detects the external secreted acidification from osteoclasts, the nanoliposomes can target the bone surface to form an alkaline protective layer, thus neutralizing the acid secretions of osteoclasts. By precisely restraining the abnormal activation of osteoclasts, the sequential initiation of osteoclast apoptosis promotion will further promote the release of extracellular vesicles. Apoptosis-derived extracellular vesicles contain receptor activator of nuclear factor-κ B (RANK), which further consumes RANK ligand (RANKL) in serum. This biological cascade could reverse bone destruction, remodel the bone microenvironment, and promote osteogenesis, offering promise as a therapeutic for osteoporosis.

Although this strategy can exert antibiotic and osteogenic effects, its osteogenic ability still needs to be further enhanced. The issue of high local concentrations of antibiotics impeding further bone restoration also needs to be addressed in the future.

### Endogenous electric field-responsive strategy

**Fig. 10:**
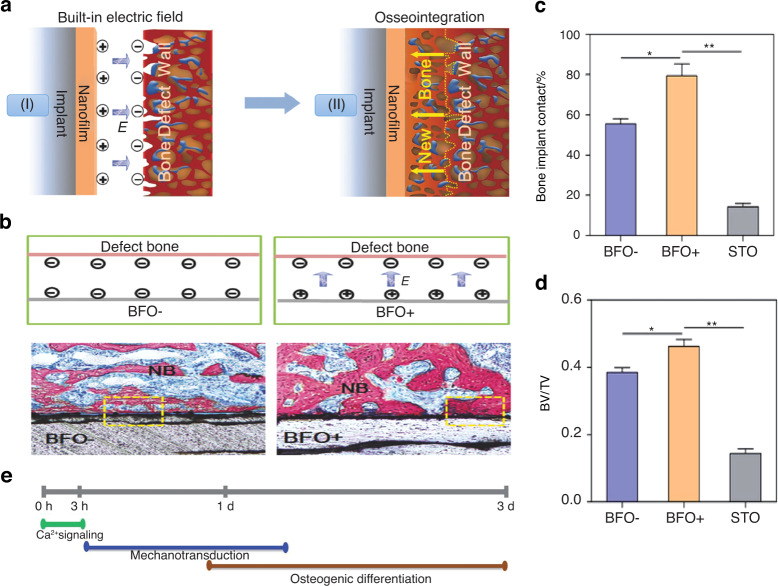
Built‐in electric fields dramatically induce enhancement of osseointegration and bone defect regeneration. **a** Illustration showing the built-in electric fields promote implant osseointegration: A built-in electric field is constructed among the endogenous electronegative bone defect wall and the electropositive ferroelectric BiFeO_3_ (BFO) nanofilm implant surface. Thus, the rapid and high-quality osseointegration was induced on the implant. **b** Schematic illustration of the built-in electric fields forming among BFO^+^ nanofilm implants and bone. And the corresponding histological analysis at 2 weeks post-implantation, which showing better osseointegration on BFO^+^ nanofilm implants (NB: nascent bone). **c** Diagram of quantitative analysis of bone-implant contact (BIC) values. **d** Diagram of different bone volume/total volume (BV/TV) on the basis of histomorphometry analysis (*P < 0.05, **P < 0.01). **e** Graphical summary of phases of BFO^+^ nanofilm-trigged osteointegration. Reproduced with permission.^28^ Copyright 2017, Wiley-VCH

External electric fields have been demonstrated to induce osteogenesis in numerous studies, which have been mentioned before. In addition, endogenous electric fields naturally exist in vivo and play a vital role in bone generation, possibly regulating cell differentiation and proliferation and thus ultimately promoting bone repair. Repairing the physiological electric microenvironment will facilitate bone damage regeneration.^112^ The endogenous electronegative potentials in bone defect sites were applied by Liu et al.^28^ to fabricate electropositive ferroelectric BiFeO_3_ (BFO) nanofilms, which could establish built-in electric fields between the electropositive BFO nanofilms and the electronegative bone defect walls, thus triggering implant osseointegration and biological healing (Fig. 10a–d). The sequential initiation of Ca^2+^ signaling, cell adhesion, and PI3K/AKT signaling in stem cells may potentially promote osteogenesis (Fig. 10e). These nanocomposite membranes have the advantages of good applicability, remarkable flexibility, and simple fabrication, which offer a well-suited and innovative strategy for bone repair. However, the long-term toxicity of the biomaterial and the long-term control of the stimulus intensity need to be examined in the future.

### Specific ionic concentration-responsive strategy

Ionic strength usually varies from one type of biological fluid to another.^113^ Specific ions will be released in a specific pathological environment. Thus, the levels of various physiological electrolytes could be critical indicators for different diseases and can be applied in a strategy to activate bone regeneration.^29^ Krishna et al.^114^ used a polyelectrolyte of CS and HA (CS-HA) to make scaffolds and loaded them with bovine serum albumin to study the release properties in response to Ca^2+^. The extent of drug release was studied in deionized water and aqueous solutions of Ca^2+^ and Na^+^, which showed rapid release of drugs increasing with the concentration of Ca^2+^. This novel smart material seems to have the potential to trigger the release of drugs or growth factors at Ca^2+^-rich sites such as bone cracks and could thus facilitate the precise and rapid repair of fractured bones. Tan et al.^115^ combined capped metal–organic frameworks (MOFs) and supramolecular pseudorotaxanes to fabricate mechanized Zr-MOFs. These novel biomaterials have the characteristics of high drug encapsulation, good biocompatibility, and low cytotoxicity. The low pH and high Ca^2+^ concentration around bone tumor cells can trigger the release of 5-fluorouracil, thus generating antitumor therapeutic effects in the specific pathological microenvironment. The regulation of Ca^2+^ and pH can also decrease adverse side effects and further promote bone regeneration in vivo.

Despite the relatively little research on ionic concentration-responsive materials, it is worth noting that electrolyte levels can be a crucial indicator for various diseases.^29^ Therefore, combining this strategy with advances in material technology could definitely introduce more encouraging paradigms for precise bone therapy and bone regeneration in the future.

### Specific enzyme-responsive strategy

Enzymes are highly specific and selective molecules that modulate numerous biological processes, such as protein expression and the formation of cellular adhesions.^29,116^ Owing to the enzymes’ varied roles in different biological processes and their vital effects in the bioactivities of the skeletal system and in bone disorders,^113,117^ research concerning smart enzyme-responsive biomaterials has attracted tremendous attention.^29^ For example, matrix metalloproteinases (MMPs) have been proven to be closely associated with tumor invasion and metastasis.^29,113^ Other pathological conditions, such as osteoarthritis, osteoporosis, and rheumatoid arthritis, are also found to involve the overexpression of MMPs in bone and cartilage cells.^117–119^ Furthermore, enzymes exhibit remarkable selectivity for their substrates, which allows biologically inspired chemical reactions to be processed specifically and sophisticatedly.^120^

Based on these properties, several smart stimuli-responsive biomaterials have been exploited, applying dysregulated enzymes as a biological trigger to achieve multiple functions, including diagnostics, drug targeting, drug release, and tissue regeneration.^120–125^ Ding et al.^126^ constructed a novel enzyme-responsive platform (LBL@MSN-Ag NPs) by loading Ag NPs in mesoporous silica NPs (MSNs) and assembling poly-L-glutamic acid (PG) and polyallylamine hydrochloride on the resulting particles by LBL assembly. In the presence of glutamyl endonuclease (V8 enzyme), which is secreted by *S. aureus* in association with virulence, PG will be degraded since it is a homogeneous polyamide made by an amide linkage. Therefore, the excess V8 enzyme in the environment of a bacterial infection would degrade PG and break down the film of LBL@MSN-Ag NPs, causing the release of loaded Ag NPs and thereby achieving excellent antimicrobial efficacy. In addition, the assembled PG in the biomaterial is a synthetic polypeptide that has outstanding biocompatibility and exhibits the potential to increase regeneration capacities. Thus, this rational smart on-demand enzyme-responsive platform can exhibit remarkable antimicrobial properties while reducing Ag ion toxicity to healthy tissue and simultaneously enhancing regeneration.

**Fig. 11:**
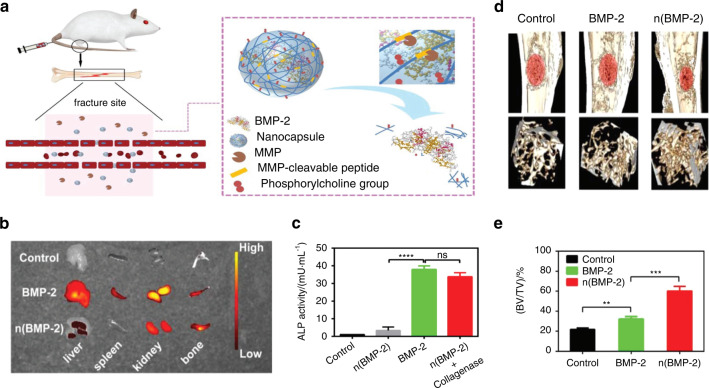
Systemic administration of enzyme-responsive growth factor nanocapsules for promoting bone repair. **a** Illustration showing the mechanism of enzyme-responsive BMP-2 nanocapsule (n(BMP-2)) and their responsive delivery for bone fracture repair. **b** The distribution of BMP-2 and n(BMP-2) in bone defect site and other tissues site after intravenous injection of BMP-2 and n(BMP-2). **c** Comparation of the expression of alkaline phosphatase (ALP) activity of MSCs with BMP-2 or n(BMP-2) incubated (****P < 0.000 1). **d** Comparation of the Micro-CT images of different therapy after rat tibial fracture. **e** Comparation of the volume of bone tissue per volume of total tissue (BV/TV) after different therapy (**P < 0.01, ***P < 0.001). Reproduced with permission.^127^ Copyright 2019, Royal Society of Chemistry

Similarly, to address the issues of the local delivery of growth factors to complex bone fracture sites, Qi et al.^127^ recently constructed BMP-2 nanocapsules (denoted as n(BMP-2)) by the in situ polymerization of an MMP-cleavable peptide crosslinker and 2-(methacryloyloxy)ethyl phosphorylcholine monomer on the surface of BMP-2 (Fig. 11a). The tissue surrounding the bone fracture site will initiate the bone repair process. Thus, MMPs, which are present only at low levels in normal tissues, are secreted at high levels into the extracellular matrix to degrade proteins. At this time, the crosslinker will be specifically degraded by MMPs (Fig. 11b). This unique reaction will trigger the destruction of the polymer shells, thus releasing BMP-2 at the fracture sites to repair bone injury and enhance bone regeneration (Fig. 11c–e). This rational design achieves outstanding osteogenic results and provides an alternative method for the rapid recovery of complex bone fractures.

Despite the encouraging results of this strategy, some troublesome problems remain to be solved in future research. First, since many similar enzyme families share overlapping substrates, more rational and specific designs should be considered for a more precise response.^120^ Second, biocompatibility and long-term cytotoxicity should be evaluated thoroughly, thus facilitating eventual clinical translation. Finally, various forms of enzyme dysregulation exist in different diseases. Thus, a comprehensive understanding of biological processes is still the basis of future research.

### Specific immune environment-responsive strategy

The highly complicated immune system comprises the synergistic action of various immune cells that can produce various cytokines.^128^ The implantation of various biomaterials and the progression of different diseases, such as bone fracture, bone infection, diabetes mellitus (DM), and even osteoporosis, can cause immune responses and changes in the immune microenvironment.^129–131^ Variations in the immune state originate from the disease and ultimately affect the progression of the disease. Accordingly, immunotherapy, which can trigger the body’s immune response to treat diseases with few side effects, has gained considerable attention in recent years, especially cancer immunotherapy.^132^ In addition to disease therapy, the immune system plays a vital role in the process of bone tissue regeneration. After implantation, the immune system can recognize implant biomaterials as “foreign” and initiate a fast response. Immune cells try to phagocytose or encapsulate the biomaterial, while inflammatory cytokines are secreted to assist this attack. Immune cells, such as T lymphocytes, B lymphocytes, neutrophils, mast cells, dendritic cells (DCs), and macrophages, participate in the central control of the formation of the local bone microenvironment. By regulating the expression of growth factors, inflammatory factors, chemokines, and other factors, immune cells regulate several processes of bone regeneration, such as cell recruitment, osteogenic differentiation, osteoclastic differentiation, vascularization, and fibrosis.^133–136^ Among all immune cells, macrophages, which can be divided into M1 type and M2 type, play vital roles in the wound healing process.^128^ M1 macrophages mainly regulate the osteoclastic process, while M2 macrophages are mainly involved in tissue repair during the middle and end stages of bone regeneration,^137^ when they secrete a variety of cytokines, such as BMP-2 and VEGF, to ultimately induce bone formation.^138^ In addition to secreting various cytokines by switching their polarization states, macrophages are responsible for the recruitment of other cells to the injury sites and for the phagocytosis of unwanted materials. Thus, macrophages play the most important role during the process of bone tissue regeneration.^128,139^ In addition to these immune cells, various cytokines are essential parts of the immune system. For example, interleukin (IL)-4 and TGF-β have been implicated in promoting osteoblast migration and proliferation. However, tumor necrosis factor (TNF)-α and IL-1β inhibit this process. Other proinflammatory cytokines, such as TNF-α and IL-1, promote osteoclast cell activity and are involved in bone loss in osteoporosis.^140,141^ All immune cells and various cytokines construct the bone immune microenvironment.^137,142^

The traditional strategy for tissue engineering was to design inert biomaterials to minimize the immune response.^143^ However, this effort failed owing to insufficient nutrient supply and poor blood vessel invasion, which ultimately led to poor regenerative outcomes.^128,136^ Currently, many researchers have realized the benefit of the immune response in tissue regeneration and found that the inflammatory response of immune cells to biomaterials is a vital factor in bone tissue regeneration.^144,145^ For example, polarization of the anti-inflammatory M2 phenotype was proven to produce a favorable osteoimmune environment, thus enhancing osteogenesis.^146^ In addition, lymphocytes contribute to osteogenesis and bone resorption. Activated T lymphocytes can express RANKL to promote the formation of osteoclasts and thus enhance bone resorption.^147^ However, B lymphocytes can also release interferon-γ to inhibit the formation of osteoclasts, thus preventing excessive bone destruction in the physiological inflammatory response.^148^ Due to this recognition, numerous strategies have been attempted to better utilize the immune system to improve the host-implant interaction. Developing a drug delivery system, exploiting novel immunomodulatory biomaterials, incorporating inflammatory cytokines, and applying novel coatings are currently effective strategies to modulate the osteoimmunomodulatory properties of biomaterials, thereby shifting the immune environment from osteoclastogenesis to osteogenesis.^128,143,149–154^

However, due to the highly complex and changeable properties of the immune environment, persistence and gentle effects on lesions could not improve bone therapy and bone regeneration results. Therefore, smart stimuli-responsive biomaterials specific to various immune microenvironments have gradually attracted increasing attention. Many chronic diseases involve long-term inflammatory bone destruction and are difficult to treat in the clinic. The development of smart specific immune environment-responsive biomaterials has become promising for therapeutics in the past half-decade. Hu et al.^155^ fabricated a novel injectable microsphere that could serve as an osteoimmunomodulatory biomaterial to modulate macrophages and create a healing-promoting environment in DM. The self-assembled microsphere incorporated heparin-modified gelatin nanofibers, and IL-4 was linked to the nanofibrous heparin-modified gelatin microsphere (NHG-MS) to serve as an immunomodulatory cytokine. Under the proinflammatory microenvironment of DM, IL-4-loaded NHG-MS can respond to proinflammatory M1 macrophages and switch them to the M2 phenotype, thus restoring the M2/M1 ratio to normal. This transformation can efficiently resolve inflammation, enhance osteoblastic differentiation, and promote new bone formation. Hence, this novel injectable microsphere represents a promising strategy to improve bone healing and resolve inflammation under DM.

In addition to chronic diseases, smart specific immune environment-responsive biomaterials would also have outstanding efficiency for patients with acute inflammation and tumors. Accordingly, drug-loaded double-layer sol–gel coatings were used to functionalize TiO_2_ nanotubes to modulate the switch from the M1 to the M2 phenotype.^156^ Under inflammatory conditions, the M0 phenotype is polarized to M1 macrophages via the release of proinflammatory cytokines. Novel smart biomaterials will respond to excess M1 macrophages and release IL-4 to directly regulate polarization from M1 to M2 macrophages, thus modulating the inflammatory response and promoting tissue repair. This novel strategy provides an idea for developing functional biomaterials to enhance tissue regeneration and change the pathological state of inflammation in lesion sites. In addition to acute inflammation, the bone metastasis of cancer is a major clinical problem, with the current treatment being severely destructive. To solve this difficult problem, He et al.^157^ recently fabricated a niobium carbide (Nb2C) MXene-modified 3D-printed biodegradable bioglass scaffold (BG@NbSiR) by loading an immune adjuvant (R837) to achieve checkpoint blockade immunotherapy. On the one hand, the loaded mesoporous Nb2C@Si NSs provided outstanding photothermal conversion performance under NIR irradiation, enhancing tumor ablation capacity. Furthermore, BG scaffolds provided valuable elements (Ca, P, Si, etc.) and sufficient space for bone regeneration. In particular, R837 offered an immune-activating vaccine-like function. In combination with the mass of tumor debris released by photonic hyperthermal ablation, R837 promoted cytokine secretion and DC recruitment/maturation, thus ultimately activating an immune response to the tumor. By utilizing checkpoint blockade immunotherapy and photonic hyperthermia, this BG@NbSiR scaffold could ablate primary tumors and activate the immune response, thus preventing tumor recurrence and metastasis.

Although initiating bone therapy and regeneration according to the site-specific immune environment of the lesion is a clever and effective strategy, several issues still need to be addressed. First, macrophages are the primary source of mediators to initiate inflammation; thus, the unrestricted activation of macrophages may damage host immune homeostasis. Furthermore, improperly polarized macrophages at the lesion site may also initiate osteoclast formation and subsequent osteolysis.^136^ Thus, the precise modulation of macrophage behaviors may be a necessary focus of future research before applying specific immune environment-responsive strategies to enhance bone tissue therapy and regeneration. Second, additional research is necessary to determine the lowest concentration of released IL-4 to induce macrophage polarization while maintaining host immune homeostasis.^136,156^

## Multiresponsive strategies for bone therapeutics and regeneration

Combination therapy can usually achieve better therapeutic outcomes than a single therapeutic modality owing to the synergistic effects of multiple therapeutic modalities.^22,158^ Similarly, multiresponsive biomaterials, which are rationally designed with multiple modalities, provide platforms not only for bone disease therapy but also for bone regeneration. To address tumor-related bone defects, a mesoporous BG/CS porous scaffold (MBCS) loaded with magnetic SrFe_12_O_19_ NPs was constructed by Lu et al.^159^ The magnetic field introduced by MBCS could promote the expression levels of osteogenic-related genes and enhance bone formation by activating the BMP-2/Smad/Runx2 pathway. In addition, the loaded SrFe_12_O_19_ NPs could improve the photothermal conversion capability and elevate the temperatures of tumors under exposure to NIR laser irradiation, which could cause apoptosis and ablation of residual tumors. Synergistic therapy combining a magnetic field and NIR laser can exert an excellent effect on tumor ablation and bone regeneration and is highly promising in the treatment of tumor-related bone deficiency. In addition, to address the challenging problem of infection after implantation, Su et al.^31^ used S-doping to create oxygen deficiencies on Ti implants, which endowed the implants with remarkable photothermal and sonodynamic abilities. Under exposure to NIR light and ultrasound treatments, implants without external antibacterial coatings achieved an antibacterial efficiency of 99.995%. Moreover, improved osseointegration was observed after the successful treatment of bone infection by combination treatments.

In addition to the novel combination of two kinds of external stimulus-responsive strategies, some researchers have attempted to combine an external stimulus-responsive strategy with an internal microenvironment stimulus-responsive strategy and have achieved outstanding results. For instance, Tan et al.^115^ designed multiresponsive “gated scaffolds” by combining supramolecular pseudorotaxanes and capped MOFs. The combination of the low pH around bone tumor cells, the Ca^2+^ concentration due to osteolysis, and hyperthermia produced a synergistic effect in cancer therapy and bone regeneration. Coincidentally, the AKT-Fe_3_O_4_-CaO_2_ scaffold was rationally designed by Dong et al.^24^ for multifunctional bone tumor therapy and bone tissue regeneration. On these smart stimuli-responsive platforms, Fe_3_O_4_ NPs function as mediators for magnetic hyperthermia for quick temperature elevation under irradiation with an alternating magnetic field. In addition, CaO_2_ NPs were loaded into the smart platform to produce sufficient H_2_O_2_ in the low-pH environment of osteolysis sites. The resulting production of H_2_O_2_ can trigger the Fenton reaction and finally induce tumor-oxidative therapy (Fig. 5a). Another product in this reaction was Ca^2+^, which has a synergistic effect with magnetic hyperthermia in bone regeneration. In addition, Ma et al.^105^ recently designed and fabricated Fe-CaSiO_3_ composite scaffolds (30CS) by the 3DP technique for synergistic tumor treatment and bone regeneration (Fig. 8a). In these smart composite scaffolds, four unique functionalities contributed to tumor therapy and remarkable bone regeneration results. First, in the intrinsically acidic tumor microenvironment, the loaded Fe-containing component could serve as a Fenton reaction nanocatalyst to trigger the decomposition of H_2_O_2_, thus causing the death of cancer cells (Fig. 8b, c). Second, the novel scaffolds exhibited excellent photothermal effects, elevating the tumor temperature under NIR irradiation. This effect also synergistically strengthened the catalytic Fenton reaction to promote ROS production. Third, these scaffolds possessed high compressive strength, providing sufficient mechanical support for new bone formation. Finally, these novel scaffolds could support the adhesion, proliferation, and differentiation of rBMSCs, thus enhancing bone regeneration in vivo (Fig. 8d). Therefore, these novel smart stimuli-responsive scaffolds are promising for the treatment of bone tumors and the regeneration of bone defects resulting from surgery.

In addition to broad application in the field of tumor therapy, multiresponsive strategies also show broad prospects for application in other fields. Recently, Zhou et al.^77^ fabricated a novel PPy-PDA-HA film by uniformly and alternately coating PPy-PDA NPs and HA NPs onto the scaffold (Fig. 7). This rational design endows the scaffold with osteoinductivity, electroactivity, antioxidative activity, and cell affinity. By responding to the excessive ROS generated by inflammation, the PPy-PDA NPs exhibited long-term antioxidative capacity to protect cells from injury. After electrical stimulation, the PPy-PDA NPs transmitted this stimulus to the cells adhering to the surface and improved cell proliferation. The synergistic effect of HA and electrical stimulation promoted osteogenic cell differentiation and exhibited remarkable bone regeneration results.

Despite the encouraging results of multiresponsive synergistic therapy, several issues still exist to address in the future. First, the existing smart multiresponsive biomaterials still combine varied strategies without adequate synergy of the fundamental mechanisms. Thus, the clinical application requirements and the relevant therapeutic mechanisms should be fully considered when designing a multiresponsive scaffold in the future. In addition, due to the complex structures and multiple compositions of smart multiresponsive biomaterials, the development of precise and convenient manufacturing processes would also be necessary in the future.

## Conclusion and future prospects

**Fig. 12:**
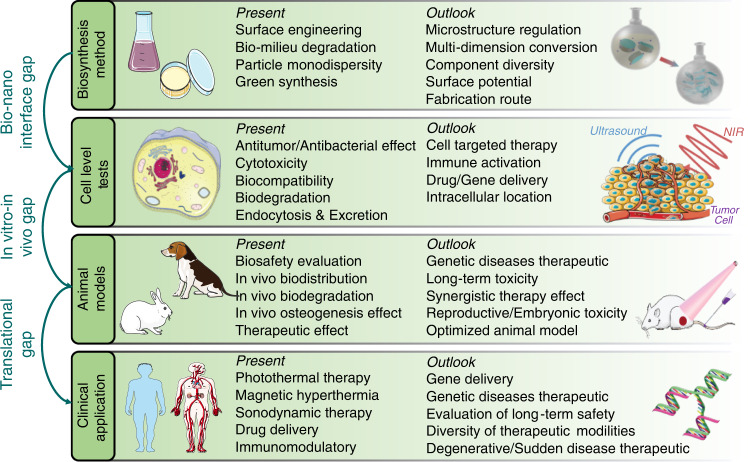
Summative scheme of the current research developments and the future outlook in smart stimuli-responsive biomaterials with multiple functions of bone therapeutics and bone regeneration

Herein, we have summarized and discussed various strategies applied in constructing unique bifunctional biomaterials for bone disease therapy and bone tissue regeneration. External physical triggers (e.g., magnetic and electric fields, ultrasound, light irradiation or appropriate mechanical stimulation) or endogenous disease microenvironments (e.g., excess ROS, mild acidity, endogenous electric fields, the immune microenvironment, specific ionic or enzyme concentrations) can be applied in the construction of smart stimuli-responsive biomaterials to achieve better therapeutic and regeneration targets. The features, advantages, and disadvantages of different responsive strategies are summarized in Table 1. After the short-term and efficient treatment of severe infection, residual tumor tissue, or other bone diseases, a therapeutic biomaterial should facilitate cell adhesion, and smart stimuli-responsive materials will thus release bioactive components or osteogenic-related elements to accelerate cell proliferation and differentiation. All these factors ultimately enhance bone regeneration.^21^ These novel biomaterials could play a vital role in precise and efficient bone disease therapy and bone tissue regeneration in the future (Fig. 12).

**Table 1:** Comparison of different response strategy types

Response strategy types	Typical methods or materials	Features and advantages	Existing problems	References
*External stimuli-responsive strategies*				
Photoresponsive strategy	Loading photothermal agents as follows: (1) gold nanostructures (2) transition metal sulfides and oxides (e.g., CuFeSe_2_ nanocrystals, Fe_3_O_4_ NPs, and copper silicate microspheres) (3) organic NPs (4) carbon-based NPs and graphene (5) MXenes and single-elemental nanosheets (e.g., black phosphorus nanosheets)	(1) noninvasive with high controllability (2) remarkable photothermal therapy effects	(1) low tissue penetration (2) intense photothermal effect may cause damage to the surrounding normal tissue (3) potential toxicity with the use of photoactivated materials	^29,47–53^
Magnetic field-responsive strategy	Loading magnetic materials or thermally sensitive agents such as Fe_3_O_4_ NPs, MnFe_3_O_4_ NPs, or Fe_3_O_4_ NPs, and so on	(1) high tissue-penetrating capabilities (2) noninvasive with high controllability (3) harmless to normal tissues	(1) the magnetic heat was not uniform (2) the high local heat could cause thermal damage to surrounding tissue	^24,61,160,161^
Ultrasound-responsive strategy	Employing the effect of ultrasound activating sonosensitizers for therapy	(1) remarkable tissue penetration depth (2) noninvasive (3) no drug resistance	(1) low in vivo stability of sonosensitizer drugs (2) potential toxicity of sonosensitizers	^27,31,62^
Electroresponsive strategy	Loading electroactive materials, such as carbon nanotubes, metal, graphene, inorganic electroactive materials, and conductive polymers	(1) improved conductive characteristics (2) remarkable tissue regeneration effect	(1) cytotoxicity, biocompatibility, and biodegradability remain uncertain (2) low control precision	^67,73,76^
Piezoelectricity-responsive strategy	Loading piezoelectric biomaterials, including piezo-bioceramics and some piezo-biopolymers	(1) improved conductive characteristics (2) remarkable regeneration effect without extraneous drugs or growth factors	(1) densification, volatilization of alkali, and high temperature in synthesis processes (2) long-term biosafety and cytotoxicity remain uncertain	^83,84,162^
Mechanical stimuli-responsive strategy	Applying proper mechanical stimulus in the regeneration platform	remarkable regeneration effect without extraneous drug or growth factors	(1) optimal mechanical parameters, such as amplitude and frequency of mechanics, are still unknown (2) noninvasive application method to applied in the processes is still needed	^96–99^
*Internal microenvironment stimuli-responsive strategy*				
Oxidative species-responsive strategy	Using excess endogenous ROS, such as peroxides, hydroxyl radical, superoxide, singlet oxygen and alpha-oxygen, as a trigger to enhance bone regeneration	(1) smart and rapid response according to the environment (2) remarkable regeneration result and therapeutical effect	(1) the small action range and short lifespan of ROS would greatly affect the stimuli effect (2) the effect will damage normal cells at the same time	^100–102^
Acidic environment-responsive strategy	Applying the strategy to respond to the mildly acidic environment in pathological conditions, such as chronic inflammation, infected environment, or tumor environment	(1) smart and rapid response to the environment (2) change the local acid environment to facilitate bone regeneration	(1) the duration of action may not be long enough for effective therapy (2) the persistent acidic environment may impede further bone regeneration	^106–109^
Endogenous electric field-responsive strategy	Response to endogenous electric fields and repairing the physiological electric microenvironment to enhance the bone regeneration	(1) smart and rapid response according to the environment (2) change the local environment to facilitate further bone regeneration	(1) the long-term toxicity of the novel biomaterial need to be lucubrated (2) the long-term control of the stimulus intensity remains uncertain	^28,112^
Specific ionic concentration-responsive strategy	Using the specific ionic concentration as a biological trigger to enhance bone regeneration	(1) rapid and smart response according to the ionic concentration (2) change the ionic concentration to facilitate bone regeneration	(1) the action duration of action may not be long enough for effective therapy (2) the stimulus intensity was not enough for effective therapy	^29,113–115^
Specific enzyme-responsive strategy	Applying the strategy to smart response to the enzyme specifically secrete in different disease statues (MMPs in tumor statues, glutamyl endonuclease in infection statues, etc.)	(1) remarkable selectivity for their substrates (2) specific and sophisticated process	(1) the overlapping substrates between similar enzyme families would affect the specificity (2) the biocompatibility and long-term cytotoxicity still need to be evaluated (3) enzyme dysregulation will affect the action time	^29,113,120^
Specific immune environment-responsive strategy	Response to different pathological immune environments by various methods such as developing drug delivery systems, exploiting novel immunomodulatory biomaterials, and applying novel coatings	(1) smart and rapid response according to the specific immune environment (2) remarkable tissue regeneration effect	(1) the unrestricted activation of macrophages may damage the host immune homeostasis (2) improperly polarized macrophages may evoke the osteoclast formation and reduce osteolysis (3) the lowest concentration of IL-4 released needs to be further confirmed	^128,136,143,149–154,156^

Despite the favorable outcomes of previous work, smart stimuli-responsive materials are still in the preliminary stage with several challenges and concerns to be addressed in future research:

1. Since these are newly synthesized biomaterials, their immune responses, metabolic pathways, and biological distribution have not been systematically explored. Multifunctional biomaterials are loaded with multiple components to implement both therapeutic and regenerative functions, making it arduous to evaluate biosafety thoroughly. In addition to the potential long-term toxicity, the strength, toughness, and other physical or chemical properties also need to be compared with those of state-of-the-art biomaterials to enable future clinical translation.
2. An appropriate biodegradation rate of novel biomaterials with multiple components is also necessary for clinical translation. After the biosafety, biocompatibility, and biodegradation of these biomaterials are fully assessed, they can ultimately be applied clinically.
3. The construction of more novel multifunctional materials that rationally integrate different therapeutic modalities and regenerative materials is still of great importance. The reported smart stimuli-responsive materials are still limited to certain specific modalities, such as photothermal ablation, magnetic hyperthermia, SDT, and nanocatalytic therapy. In addition to these treatment models, novel biomaterials fabricated in the future could incorporate various NPs to improve bone regeneration efficiency and deliver drugs or related genes in a controllable mode for precise bone disease therapeutics. The synthesis of composite material systems utilizing newly developed therapeutic nanoplatforms and biomaterial platforms will continue to be the main direction of future research.
4. Owing to the complex fabrication processes of smart stimuli-responsive materials, the exploitation of facile synthetic methodologies to replace the existing complex synthetic procedures is indispensable. To endow these smart stimuli-responsive materials with multiple functions, researchers integrate various components into one biomaterial platform, which is implemented by several difficult procedures. To address this crucial issue, the exploitation of facile integrated methodologies is essential.
5. Various strategies, such as external stimuli-responsive or internal microenvironment stimuli-responsive approaches, have pros and cons in practical biomedical applications. The effects of the existing newly synthesized biomaterials in bone therapy and regeneration still cannot be precisely and rationally controlled. Specifically, the precise confirmation of optimum parameters for external stimuli and the rapid recognition of internal environmental changes are still difficult. Only by determining the optimum parameters, such as the depth and intensity of infrared light, can these novel strategies be finally applied in the clinic. Thus, this aspect is a definite long-term research focus.
6. The specific mechanisms of smart stimuli-responsive materials remain to be investigated in detail, requiring the selection of appropriate animal models for mechanistic studies and performance assessments. Therefore, the selection of appropriate animal models and further mechanistic exploration are still of great importance for promoting the clinical applications of these novel biomaterials with both bone therapy and tissue regeneration functions.

In conclusion, smart multifunctional stimuli-responsive materials have been explored to some extent and have received considerable attention in antibiotic therapy, tumor therapy, the prevention of inflammation, and the stimulation of tissue repair. Although some challenges still exist and there is a long way to go for clinical translation, it is expected that smart stimuli-responsive materials will have profound biomedical applications in the future.

## Supplementary information

Response to reviewer

Licenses for the Cited Figs. 2–11
