# Supplementary material for: Recent advances in smart stimuli-responsive biomaterials for bone therapeutics and regeneration
Source: Bone Res. 2022 Feb 23;10:17. doi: 10.1038/s41413-021-00180-y (PMC8866424; doi:10.1038/s41413-021-00180-y)
Supplement: Supplementary file 1 — Response to reviewer [file 41413_2021_180_MOESM1_ESM.docx]

**Responses to Comments**

**Reviewer 1:**

**Comments from reviewer 1:**

As indicated in my comments to the authors, the revisions are largely cosmetic. The requested discourse about immunomodulation is unsatisfactory as is the "correction" of linguistic shortcomings, that was requested from all three reviewers. The many errors and mistakes seriously distract from the message of the paper. At this stage, I suggest that this manuscript should not only be checked by the authors but copyedited by a native English speaker.

In this revised version of their review paper, the authors have tried and partially succeeded in responding to the constructive criticism of this reviewer. On the plus side, they succeeded in more clearly distinguishing their review of “smart biomaterials” from that on the same topic recently published in the same journal.

By contrast, modifications to the text in response to this reviewer’s second query (immune-responsive materials) remain mostly superficial - except, perhaps, in the section on “elaboration of the immune environment”. As to the other revisions, simply changing or adding a word here and there does not enhance the clarity and the depth of the discussion. This reviewer would expect a more in-depth survey of the use of immunomodulatory biomaterials in hard-tissue engineering and for therapeutic purposes.

The authors were even less successful in correcting the linguistic problems that were unanimously pointed out by all three reviewers. The 19 suggested “amendments” represent mostly incremental minute changes that do not amount to substantial clarification or improvement, moreover they often leave existing grammatical/linguistic errors or introduce new ones. The authors claim to have checked their manuscript using Grammarly. Nevertheless, when this reviewer checked the revised version of the manuscript using the open-access version of Grammarly, the software revealed more than 300 errors, some of them minor in nature, related to punctuation, but quite a large number more serious (e.g., missing indefinite or definite articles, awkward English phrases, etc.). These linguistic mistakes significantly detract from the otherwise interesting contents of this review. Even after correcting the problems identified by Grammarly, the entire manuscript should be copy-edited by a native English speaker for linguistic correctness.

***Response:*** Thank you very much for the positive comment and recommendation. According to the suggestions, the following revisions have been made:

**1.** **“By contrast, modifications to the text in response to this reviewer’s second query (immune-responsive materials) remain mostly superficial - except, perhaps, in the section on “elaboration of the immune environment”. As to the other revisions, simply changing or adding a word here and there does not enhance the clarity and the depth of the discussion. This reviewer would expect a more in-depth survey of the use of immunomodulatory biomaterials in hard-tissue engineering and for therapeutic purposes”**

***Response****:* Thank you very much for the constructive suggestion. According to the reviewer’s suggestion, the corresponding descriptions and discussions have been supplemented in the revised manuscript.

1) The sentence (Page 40, Line 19-20 and Page 41, Line 1 in original manuscript) **‘The highly complicated immune system comprises the synergistic action of various immune cells that can produce various cytokines.’** has been moved to beginning of this section (Page 40, Line 9-10 in the revised manuscript) to summarize the composition of immune system.
2) The sentence (Page 40, Line 13-17 in the revised manuscript) **‘Variations in the immune state originate from the disease and ultimately affect the progression of the disease. Accordingly, immunotherapy, which can trigger the body’s immune response to treat disease with few side effects, has gained considerable attention in recent years, especially cancer immunotherapy. In addition to disease therapy, the immune system also plays a vital role in the process of bone tissue regeneration.’** have been added in Section 3.6 to supplement the elaboration of the effect of immune system in the progression of the disease.
3) The sentence (Page 38, Line 14-17 in original manuscript) **‘After implantation, the immune system can recognize the implants biomaterials as ‘foreign’ and initiate a fast response. Immune cells try to phagocytose or encapsulate the biomaterial, while inflammatory cytokines are secreted to assist this attack.’** has been changed the location (Page 40, Line 18-21 in the revised manuscript) to start the introduction of the immune response after implantation.
4) The sentence (Page 40, Line 21 and Page 41, Line 1-6 in the revised manuscript) **‘Immune cells, such as T lymphocytes, B lymphocytes, neutrophils, mast cells, dendritic cells and macrophages, participate in the central control of the formation of the local bone microenvironment. By regulating the expression of growth factors, inflammatory factors, chemokines and other factors, immune cells regulate several processes of bone regeneration, such as cell recruitment, osteogenic differentiation, osteoclastic differentiation, vascularization, and fibrosis.’** have been added in Section 3.6 to supplement the elaboration of the role of immune cell after implantation.
5) Among all immune cells, macrophages play a vital role in bone tissue engineering. Thus, the effect of macrophages after implantation has been illustrated and discussed in detail in the revised manuscript as followings (Page 41, Line 6-15 in the revised manuscript):

**“Among all immune cells, macrophages, which can be divided into M1 type and M2 type, play a vital role in the wound healing process. M1 macrophages mainly regulate the osteoclastic process, while M2 macrophages are mainly involved in tissue repair during the middle and end stages of bone regeneration, when they secrete a variety of cytokines, such as BMP-2 and VEGF, to ultimately induce bone formation. In addition to secreting various cytokines by switching their polarization states, macrophages are also responsible for the recruitment of other cells to the injury sites and for the phagocytosis of unwanted materials. Thus, macrophages play the most important role during the process of bone tissue regeneration.”**

6) The sentence (Page 41, Line 15-16 in the revised manuscript) **‘In addition to these immune cells, various cytokines are also essential parts of the immune system.’** have been added for the transition of the follow introduction of cytokines.
7) The sentence (Page 41, Line 20-21 in the revised manuscript) **‘All the immune cells and various cytokines construct the bone immune microenvironment.’** have been added for proposing new concept of bone immune microenvironment.
8) To further supplement the new concept of bone immune microenvironment. More detail elaboration of the effect of lymphocyte after implantation was added in the revised manuscript (Page 42, Line 8-13 in the revised manuscript):

**“In addition, lymphocytes also contribute to osteogenesis and bone resorption. Activated T lymphocytes can express RANKL to promote the formation of osteoclasts and thus enhance bone resorption147. However, B lymphocytes can also release interferon⁃γ (IFN⁃γ) to inhibit the formation of osteoclasts, thus preventing excessive bone destruction in the physiological inflammatory response.”**

9) The sentence (Page 43, Line 3-6 in the revised manuscript) **‘Many chronic diseases involve long-term inflammatory bone destruction and are difficult to treat in the clinic. The development of smart specific immune environment-responsive biomaterials has become an encouraging therapeutic in the past half-decade.’** have been added for the transition of the follow introduction of the application in chronic diseases.
10) Besides chronic diseases, the smart specific immune environment-responsive biomaterials can also be applied in some acute inflammation. Thus, the sentence (Page 43, Line 18-20 in the revised manuscript) **‘In addition to chronic diseases, smart specific immune environment-responsive biomaterials would also have outstanding efficiency for patients with acute inflammation and tumors.’** have been added for the transition for introducing the application of smart specific immune environment-responsive biomaterials in acute inflammation and tumors.
11) Bone metastasis is also a major clinical problem, the smart specific immune environment-responsive biomaterials can also be applied to solve this difficult problem. Thus, a typical case published in recent research was added in the revised manuscript (Page 44, Line 7-22 in the revised manuscript):

**“In addition to acute inflammation, bone metastasis of cancer is also a major clinical problem, with the current treatment being severely destructive. To solve this difficult problem, He et al.157 recently fabricated a niobium carbide (Nb2C) MXene-modified 3D-printed biodegradable bioglass scaffold (BG@NbSiR) by loading an immune adjuvant (R837) to achieve checkpoint blockade immunotherapy. On the one hand, the loaded mesoporous Nb2C@Si NSs provided outstanding photothermal conversion performance under NIR irradiation, enhancing tumor ablation capacity. Furthermore, BG scaffolds provided valuable elements (Ca, P, Si, etc.) and sufficient space for bone regeneration. In particular, R837 offered an immune-activating vaccine-like function. In combination with the mass of tumor debris released by photonic hyperthermal ablation, R837 promoted cytokine secretion and dendritic cell (DC) recruitment/maturation, thus ultimately activating the immune response to the tumor. By utilizing checkpoint blockade immunotherapy and photonic hyperthermia, this BG@NbSiR scaffold could ablate primary tumors and activate the immune response, thus preventing tumor recurrence and metastasis.”**

**2. “The authors were even less successful in correcting the linguistic problems that were unanimously pointed out by all three reviewers. The 19 suggested “amendments” represent mostly incremental minute changes that do not amount to substantial clarification or improvement, moreover they often leave existing grammatical/linguistic errors or introduce new ones. The authors claim to have checked their manuscript using Grammarly. Nevertheless, when this reviewer checked the revised version of the manuscript using the open-access version of Grammarly, the software revealed more than 300 errors, some of them minor in nature, related to punctuation, but quite a large number more serious (e.g., missing indefinite or definite articles, awkward English phrases, etc.). These linguistic mistakes significantly detract from the otherwise interesting contents of this review. Even after correcting the problems identified by Grammarly, the entire manuscript should be copy-edited by a native English speaker for linguistic correctness.”**

***Response****:* Thank you very much for the constructive suggestion. According to the reviewer’s suggestion, we reviewed the manuscript and corrected some language mistakes. After that, the manuscript was sent to the SPRINGER NATURE author services and edited for proper English language, grammar, punctuation, spelling and overall style by the highly qualified native English-speaking editors. The Editing certificate was provided in the supplementary material. All changes are highlighted in yellow in the marked copy of our manuscript for your reference. And some major adjustments have also been listed below:

1) The sentence (Page 2, Line3-4 in original manuscript) **‘Autologous and allogeneic grafts are two main traditional remedies for it, which could cause series of complications.’** has been revised as (Page 2, Line5-6 in the revised manuscript) **‘Autologous and allogeneic grafts are two main traditional remedies, but they can cause a series of complications.’**
2) The sentence (Page 2, Line7-12 in original manuscript) **‘Thus, the rational design and integration of versatile biomaterials with bone therapeutics and regeneration functions are in great demand. Many strategies, including external physical triggers stimulus, endogenous disease microenvironments stimulus, or an integration of multiple strategies, had been applied to fabricate smart stimuli-responsive materials for bone therapies and regenerations.’** has been revised as (Page 2, Line9-13 in the revised manuscript) **‘Thus, the rational design of versatile biomaterials with integrated bone therapy and regeneration functions is in great demand. Many strategies have been applied to fabricate smart stimuli-responsive materials for bone therapy and regeneration, with stimuli related to external physical triggers, endogenous disease microenvironments, or multiple integrated strategies.’**
3) The sentence (Page 4, Line10-12 in original manuscript) **‘The requirements of these implantable scaffolds are to match the properties of the original tissues, such as osteoinductivity, biocompatibility, osteoconductivity and suitable mechanical strength.’** has been revised as (Page 4, Line11-12 in the revised manuscript) **‘These implantable scaffolds are required to match the properties of the original tissues, such as osteoinductivity, biocompatibility, osteoconductivity and suitable mechanical strength.’**
4) The sentence (Page 4, Line18-19 in original manuscript) **‘Moreover, the implant fractures caused by inflammation, loosening, and osteolysis were still a tricky issue that remained to be settled.’** has been revised as (Page 4, Line19-20 in the revised manuscript) **‘Moreover, implant fractures caused by inflammation, loosening, and osteolysis are still challenges that remain to be addressed.’**
5) The sentence (Page 6, Line2-9 in original manuscript) **‘Based on this, quite a few of novel materials with smart stimuli-responsive bifunctions of both therapeutic and regeneration abilities were developed, which could manifest reversible or irreversible transformations in physical performances or chemical structures when responding to external physical triggers (e.g. light irradiation, electric and magnetic fields, ultrasound, proper mechanical stimulus), endogenous disease microenvironments (e.g. overexpressed ROS, mild acidity, endogenous electric fields, specific ionic concentration, secreted enzyme or specific immune environment) or an integration of them.’** has been revised as (Page 6, Line4-11 in the revised manuscript) **‘Based on these concepts, a number of novel bifunctional materials with smart stimuli-responsive therapeutic and regenerative abilities were developed31,32, which could undergo reversible or irreversible transformations in physical performance or chemical structure in response to external physical triggers (e.g., light irradiation, electric and magnetic fields, ultrasound, appropriate mechanical stimulus), endogenous disease microenvironments (e.g., overexpressed ROS, mild acidity, endogenous electric fields, specific ionic concentrations, secreted enzymes or specific immune environments) or a combination of the above.’**
6) The sentence (Page 11, Line15-18 in original manuscript) **‘Radiation under infrared laser could generate photo-physical effects, thus could subsequently change the intracellular behavior and influence the respiratory chain, which would ultimately increase ATP regeneration in the mitochondrial membrane and raise the cell metabolism.’** has been revised as (Page 10, Line15-18 in the revised manuscript) **‘Radiation under an infrared laser can generate photophysical effects, subsequently changing intracellular behavior and influencing the respiratory chain, which ultimately increases ATP regeneration in the mitochondrial membrane and heightens cell metabolism.’**
7) The sentence (Page 11, Line19-21 in original manuscript) **‘Therefore, the photo-responsive strategy is widely applied, no matter in the anti-tumor areas or anti-bacteria areas, due to its easily synthesized and the presence of plentiful photo-responsive functional nanosystems or components.’** has been revised as (Page 10, Line19-21 in the revised manuscript) **‘Therefore, the photoresponsive strategy is widely applied in both antitumor and antibacterial treatment due to its easy synthesis and the presence of plentiful photoresponsive functional nanosystems and components.’**
8) The sentence (Page 12, Line20-21 in original manuscript) **‘Under the irradiation of 808-nm NIR, the temperature can rise to 48 ℃, which can ablate human osteosarcoma cells effectively.’** has been revised as (Page 11, Line19-21 in the revised manuscript) **‘Under 808-nm NIR irradiation, the temperature was increased to 48 ℃, which effectively ablated human osteosarcoma cells.’**
9) The sentence (Page 13, Line11-14 in original manuscript) **‘Zeng et al. fabricated a biocompatible polydopamine (PDA)-IR820-Daptomycin (DAP) coating in titanium implants, which can possess triple therapy functions of antibiotic, photodynamic and photothermal.’** has been revised as (Page 13, Line3-5 in the revised manuscript) **‘Zeng et al. fabricated a biocompatible polydopamine (PDA)-IR820-daptomycin (DAP) coating in titanium implants, which possesses the triple therapeutic functions of antibiotic, photodynamic and photothermal activity.’**
10) The sentence (Page 15, Line5-7 in original manuscript) **‘The low tissue-penetrating depth of light severely limits the photothermal-conversion performance against regeneration deep in the body.’** has been revised as (Page 14, Line9-10 and Page 15, Line1 in the revised manuscript) **‘The low tissue-penetration depth of light severely limits the ability of photothermal conversion, thus would ultimately to impede the deep tissue regeneration in the body.’**
11) The sentence (Page 16, Line2-4 in original manuscript) **‘Moreover, the magnetic field-responsive therapy also shows features of noninvasive and high controllability, which are of great demand in bone tumor ablation and local bone regeneration.’** has been revised as (Page 16, Line9-11 in the revised manuscript) **‘Moreover, magnetic field-responsive therapy has the advantages of noninvasiveness and high controllability, which are of great demand in bone tumor ablation and local bone regeneration.’**
12) The sentence (Page 17, Line13-15 in original manuscript) **‘Besides that, the continuous release of Ca^2+^ within the scaffolds improves osteogenesis obviously on the basis of both micro-CT reconstruction and quantitative analysis of Van Gieson’s (VG) staining techniques.’** has been revised as (Page 17, Line5-7 in the revised manuscript) **‘In addition, the continuous release of Ca^2+^ within the scaffolds obviously improved osteogenesis, as shown by both micro-CT reconstruction and quantitative analysis of Van Gieson’s (VG) staining techniques.’**
13) The sentence (Page 18, Line10-12 in original manuscript) **‘Although all the researches related to the magnetic field triggering bone therapeutics and regeneration are at a preliminary stage, the encouraging results are still capturing more and more attention.’** has been revised as (Page 17, Line15-17 in the revised manuscript) **‘Although all the studies related to magnetic field-triggered bone therapy and regeneration are at a preliminary stage, the encouraging results are capturing increasing attention.’**
14) The sentence (Page 23, Line5-7 in original manuscript) **‘Besides, these unique coating also performed remarkable antibacterial properties, which increased 1000 times compared to pure titanium.’** has been revised as (Page 23, Line5-7 in the revised manuscript) **‘In addition, these unique coatings exhibited remarkable antibacterial properties, 1000 times greater than those of pure titanium.’**
15) The sentence (Page 26, Line19-21 and Page 27, Line1-2 in original manuscript) **‘In addition, the PVFT layer can present with the negative pole and gather positive Ca^2+^ from bioactive glass, which could activate the CaSR of osteoblasts and ultimately enhance osteogenesis. This unique work provides a novel choice in applying biomimetic piezoelectric behavior for efficient critical-sized bone regeneration.’** has been revised as (Page 25, Line21-22 and Page 26, Line1-2 in the revised manuscript) **‘In addition, the negative pole of the PVFT layer can gather positive Ca^2+^ from bioactive glass, activating the CaSR of osteoblasts and ultimately enhancing osteogenesis. This unique work presents a novel choice in applying biomimetic piezoelectric behavior for efficient bone regeneration in critical-sized bone defects.’**
16) The sentence (Page 27, Line6-7 in original manuscript) **‘However, some restrictions still remained to be addressed, such as densification, volatilization of alkali, and high temperature in synthesizing processing.’** has been revised as (Page 26, Line6-7 in the revised manuscript) **‘However, some limitations still remain to be addressed, such as the effects of densification, alkali volatilization, and high temperature in the synthesis processing.’**
17) The sentence (Page 29, Line7-9 in original manuscript) **‘Meanwhile, discovering a non-invasive method to apply in the regeneration processes is also an impendency before utilizing this method in bone tissue regeneration.’** has been revised as (Page 28, Line5-7 in the revised manuscript) **‘Moreover, discovering a noninvasive method to apply mechanical stimuli in the regeneration process is also necessary before this method can be utilized in bone tissue regeneration.’**
18) The sentence (Page 30, Line13-16 in original manuscript) **‘Thus, the excess endogenous ROS is usually applied as a trigger in the stimulation of bone therapeutics and regeneration. A multitude of researches recently have focused on this strategy and synthesized multifunctional biomaterial applying this strategy.’** has been revised as (Page 29, Line10-13 in the revised manuscript) **‘Thus, excess endogenous ROS are often used as a trigger in stimuli-responsive bone therapy and regeneration. A multitude of recent studies have focused on this strategy and synthesized multifunctional biomaterials applying this strategy.’**
19) The sentence (Page 33, Line8-10 in original manuscript) **‘Although such strategy has an effect on the improvement of antibiosis and osteogenesis, their osteogenic abilities still need to be further enhanced.’** has been revised as (Page 34, Line1-2 in the revised manuscript) **‘Although this strategy can exert antibiotic and osteogenic effects, its osteogenic ability still needs to be further enhanced.’**
20) The sentence (Page 37, Line13-16 in original manuscript) **‘Owing to the varied roles of enzymes in different biological processes and the vital effects of enzyme in the bioactivities of skeletal system with bone disorders, the research concerning smart enzyme-responsive biomaterials have caught tremendous attention nowadays.’** has been revised as (Page 37, Line7-10 in the revised manuscript) **‘Owing to the enzymes’ varied roles in different biological processes and their vital effects in the bioactivities of the skeletal system and in bone disorders, research concerning smart enzyme-responsive biomaterials has attracted tremendous attention.’**
21) The sentence (Page 37, Line 20 and Page 38, Line 1-2 in original manuscript) **‘Furthermore, the enzymes can present with remarkable selectivity for their substrates, which allow the biologically inspired chemical reactions processed specifically and sophisticatedly.’** has been revised as (Page 37, Line14-16 in the revised manuscript) **‘Furthermore, enzymes exhibit remarkable selectivity for their substrates, which allows biologically inspired chemical reactions to be processed specifically and sophisticatedly.’**
22) The sentence (Page 38, Line 9-11 in original manuscript) **‘After *S. aureus* secretes glutamyl endonuclease (V8 enzyme) and induces the virulence of *S. aureus* infection, the PG will be degraded by the V8 enzyme since it is a homogeneous polyamide made by an amide linkage.’** has been revised as (Page 38, Line 2-4 in the revised manuscript) **‘In the presence of glutamyl endonuclease (V8 enzyme), which is secreted by *S. aureus* in association with virulence, the PG will be degraded since it is a homogeneous polyamide made by an amide linkage.’**
23) The sentence (Page 39, Line 4-5 in original manuscript) **‘Thus, the MMPs, which were low in content around the normal tissues, will largely secrete into the extracellular matrix to degrade the protein.’** has been revised as (Page 38, Line 17-18 in the revised manuscript) **‘Thus, MMPs, which are present only at low levels in normal tissues, would be secreted at high levels into the extracellular matrix to degrade proteins.’**
24) The sentence (Page 40, Line 13-15 in original manuscript) **‘Finally, various enzyme dysregulation activities exist in different diseases. Thus the comprehensive understanding of the biological process is still the basis of the research in the future.’** has been revised as (Page 40, Line 6-7 in the revised manuscript) **‘Finally, various forms of enzyme dysregulation exist in different diseases. Thus, a comprehensive understanding of biological processes is still the basis of future research.’**
25) The sentence (Page 43, Line 16-19 in original manuscript) **‘Secondly, additional researches are necessary for determining the lowest concentration of IL-4 released, which can both induce macrophage polarization properly and maintain the host immune homeostasis.’** has been revised as (Page 45, Line 9-11 in the revised manuscript) **‘Second, additional research is necessary to determine the lowest concentration of released IL-4 to induce macrophage polarization while maintaining host immune homeostasis.’**
26) The sentence (Page 44, Line 1-3 in original manuscript) **‘Synergistic therapy usually can achieve more ideal therapeutic consequences than the single therapeutic modality due to the synergistic effects of multiple therapeutic modalities.’** has been revised as (Page 45, Line 14-16 in the revised manuscript) **‘Combination therapy can usually achieve better therapeutic outcomes than a single therapeutic modality due to the synergistic effects of multiple therapeutic modalities.’**
27) The sentence (Page 47, Line 2-4 in original manuscript) **‘Firstly, the existing smart multi-responsive biomaterial is still on the basis of combining varied strategies without adequate synergy on fundamental mechanisms.’** has been revised as (Page 48, Line 13-15 in the revised manuscript) **‘First, the existing smart multi-responsive biomaterials still combine varied strategies without adequate synergy of the fundamental mechanisms.’**
28) The sentence (Page 49, Line 15-18 in original manuscript) **‘Besides these remedial models, the novel-fabricated biomaterial in the future would incorporate various nanoparticles to improve bone regeneration efficiency and deliver the drugs or related genes in a controllable mode for precise bone disease therapeutics.’** has been revised as (Page 51, Line 11-14 in the revised manuscript) **‘In addition to these treatment models, novel biomaterials fabricated in the future could incorporate various NPs to improve bone regeneration efficiency and deliver drugs or related genes in a controllable mode for precise bone disease therapeutics.’**
29) The sentence (Page 50, Line 3-6 in original manuscript) **‘To endow multifunctions of these smart stimuli-responsive materials, the researchers integrate various components into one biomaterial platform, which are implemented by several procedures with great difficulties.’** has been revised as (Page 51, Line 19-21 in the revised manuscript) **‘To endow these smart stimuli-responsive materials with multiple functions, researchers integrate various components into one biomaterial platform, which is implemented by several difficult procedures.’**
30) The sentence (Page 50, Line 9-13 in original manuscript) **‘The existing new-synthesized biomaterial still cannot be precisely and rationally controlled to act in bone therapeutics and regenerations. To be specific, the precisely confirmation of optimum parameters in external stimulus and the rapid recognition of internal environmental changes are still difficult nowadays.’** has been revised as (Page 52, Line 5-8 in the revised manuscript) **‘The effects of the existing newly synthesized biomaterials in bone therapy and regeneration still cannot be precisely and rationally controlled. Specifically, the precise confirmation of optimum parameters for external stimuli and the rapid recognition of internal environmental changes are still difficult.’**

**Supplementary：Editing Certificate**
